# *Acanthamoeba* ATP synthase structure reveals the TCA cycle is tethered to OXPHOS

**DOI:** 10.1016/j.cell.2026.08.057

**Published:** 2026-10-01

**Authors:** Michelle Y. Fry, Bridget E. Luce, Jonathan A. Stefely, Michael Z. Chen, Felicia G. Deng, Tiana M. Issa, MitoCarta Tree of Life Consortium, Sarah E. Calvo, John Samuelson, Vamsi K. Mootha, Luke H. Chao

**Affiliations:** 1 Department of Molecular Biology, Massachusetts General Hospital, Boston, MA 02114, USA; 2 Department of Genetics, Blavatnik Institute, Harvard Medical School, Boston, MA 02115, USA; 3 Harvard Medical School, Boston, MA 02115, USA; 4 Broad Institute of MIT and Harvard, Cambridge, MA 02412, USA; 5 Howard Hughes Medical Institute, Massachusetts General Hospital, Boston, MA 02114, USA; 6 Department of Molecular and Cell Biology, Boston University Goldman School of Dental Medicine, Boston, MA 02118, USA

## Abstract

Oxidative phosphorylation (OXPHOS) is a key metabolic process that couples redox energy to ATP production. While some core OXPHOS complex subunits are found across all domains of life, many have diverged or expanded across evolution—as seen in the protozoan pathogen *Acanthamoeba castellanii*. By integrating cryo-electron microscopy of unenriched mitochondrial lysate with mass spectrometry proteomics, we resolved the structures of endogenous mitochondrial ATP synthase (complex V), Hsp60, and respiratory complex III from *Acanthamoeba*. We capture *Acanthamoeba* ATP synthase in an IF1-inhibited state and reveal how *Acanthamoeba*-specific subunits and extensions stabilize the molecular machine, including a β subunit extension that interfaces with the peripheral stalk. Additionally, we characterize an active malate dehydrogenase (MDH) dimer structurally integrated within the ATP synthase peripheral stalk, revealing a direct protein tether between OXPHOS and the tricarboxylic acid cycle. Together, these findings provide structural insight into lineage-specific adaptations in *Acanthamoeba* that may tune protozoan metabolism.

## Graphical Abstract

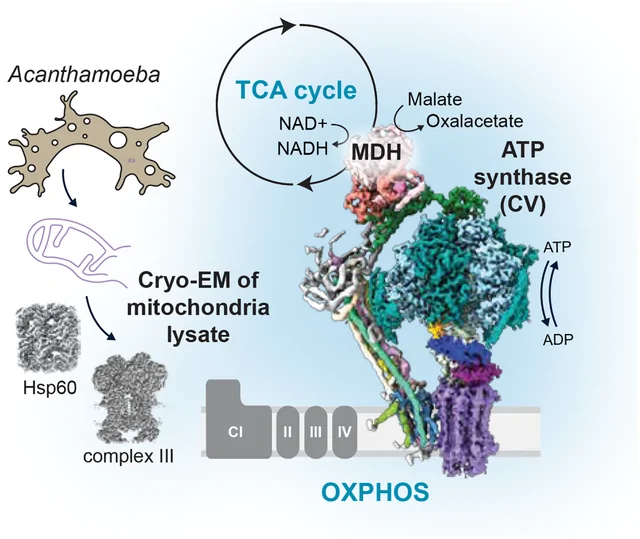

## INTRODUCTION

The mitochondrion is the site of several critical bioenergetic functions, including ATP synthesis by oxidative phosphorylation (OXPHOS) and the tricarboxylic acid cycle (TCA)^1,2^. OXPHOS complexes I–IV in the electron transport chain (ETC) couple redox reactions to generate an electrochemical gradient across the inner membrane. Translocation of protons from the intermembrane space (IMS) back to the mitochondrial matrix through ATP synthase (also known as complex V), catalyzes the formation of ATP from ADP^3^. OXPHOS and the TCA cycle are directly coupled—NADH is a product of the TCA cycle and substrate of complex I and complex II (succinate dehydrogenase, SDH) is a shared component of both processes^4^. Yet, the full extent to which the TCA cycle and OXPHOS are intertwined across eukaryotes has not been fully investigated^5^.

ATP synthase is essential for aerobic life across all domains of biology, with homologs found in bacteria, archaea, and eukaryotic organelles such as mitochondria and chloroplasts^3,6–8^. ATP synthase consists of two functional regions: the ATP synthesizing F_1_ catalytic head^9–11^, containing three alternating α and β subunits, and a membrane-embedded F_o_ domain that translocates protons across the membrane^7,11–14^. The F_1_ and F_o_ regions are connected by central and peripheral stalks, coupling rotary motion driven by proton transport to ATP synthesis.^11^ Mitochondrial ATP synthases assemble into oligomers with the dimeric form seen as the minimal functional unit^3^.

While the core subunits α, β, γ, ε, δ, c, ATP6, b, d, and OSCP are highly conserved, ATP synthases have diversified across organisms through the acquisition of lineage-specific subunits that tune regulation, stability, and oligomerization^15–18^. Many of these additional proteins contribute to the formation of dimers and higher-ordered oligomers of mitochondrial ATP synthases^19–21^. Due to the limited structural diversity captured to date, the functional and evolutionary significance of lineage-specific ATP synthase subunits remains poorly understood. While structures have been resolved from mammalian, fungal, and a few protozoan systems, many branches of the eukaryotic tree of life are unexplored^15,22–29^.

One structurally unexplored branch is the Amoebozoa eukaryotic supergroup, an evolutionary “cousin” clade to the Opisthokonta supergroup, which includes animals and fungi (Figure 1A). Both amoebozoans and opisthokonts are also members of the taxonomic supergroup Amorphea (Figure 1A)^30–32,32–34^. The extent to which ATP synthase composition, assembly, and mechanism is conserved within Amorphea is underexplored. *Acanthamoeba castellanii* is a free-living pathogenic amoebozoan that causes amoebic keratitis and fatal brain infections in humans^35^. Its mitochondria have been previously reported to exhibit ETC features not seen in humans, such as an alternative oxidase (AOX)^36–41^. Through our MitoCarta Tree of Life (MitoTOL) project, we redefined the entire proteome (via new genome reannotation in Stefely et al, co-submitted^42^), generated a high-quality mitochondrial proteome of *Acanthamoeba castellanii* (a resource called the AcMitoCarta), and cataloged conserved and lineage-specific subunits for OXPHOS complexes I–V using multiple mass-spectrometry approaches (Stefely et al co-submitted^43^). Previous work has also identified OXPHOS subunits using BN-PAGE-MS^44^, yet, the molecular architecture of these OXPHOS machines remains unknown. In particular, it is unknown how conserved and lineage-specific ATP synthase subunits are assembled in this lineage and whether amoebozoans possess distinct structural adaptations.

One notable observation from BN-PAGE-MS that has been previously reported^44^ and again seen^43^, is a malate dehydrogenase (MDH) migrating with other ATP synthase subunits. This is surprising because MDH is an integral component of the TCA cycle whereas ATP synthase is part of a separate bioenergetic process, OXPHOS^45,46^. Whether the MDH is integrated into ATP synthase or simply co-migrates as a part of another structure is unknown. To address this, we sought to solve the structure of ATP synthase from *Acanthamoeba*. Structures of mammalian, fungal, and protist (including *Trypanosoma*, *Toxoplasma*, and *Polytomella*) ATP synthases have revealed not only how lineage-specific subunits and features are assembled into the full complexes, but also provide mechanistic insight into their contribution to stabilization and function^15,22–29^.

Here we present cryo-EM structures of multiple endogenous *A. castellanii* mitochondrial protein complexes from mitochondrial lysate. We report the structures of ATP synthase, mitochondrial Hsp60 and respiratory complex III. The structure of ATP synthase reveals lineage-specific biology, extensions, and organization of subunits including an MDH integrated into the peripheral stalk. This finding reveals a structural link between OXPHOS and the TCA cycle in *A. castellanii* mitochondria. We find that integration of MDH into ATP synthase via an *Acanthamoeba*-specific scaffolding protein increases the working substrate concentration range of this TCA enzyme. This report of a metabolic TCA enzyme physically integrated into an ATP synthase expands our view of the functional and structural integration of OXPHOS and the TCA cycle in eukaryotes. Together, with comparative analyses of other mitochondrial ATP synthases, this work provides new insight into the evolution of ATP synthase assembly and oligomerization.

## RESULTS

### Determining cryo-EM structures of complexes from mitochondrial lysate

To determine the structure of mitochondrial complexes from *Acanthamoeba castellanii*, we imaged native mitochondrial lysate using an enrichment- and purification-free approach to circumvent the limited genetic tools and scalability of the pathogen^35^. Mitochondria were isolated from *Acanthamoeba* trophozoites using differential centrifugation and gently lysed in a 0.5% n-dodecyl-β-D-maltoside (DDM) buffer. Solubilized mitochondrial membranes were then vitrified on cryo-electron microscopy (cryo-EM) grids (Figure 1B). This approach did not involve purification of or enrichment of a specific target^47–51^, but rather direct imaging of a solubilized membrane mixture. Similar purification free methods have recently been used for structure determination of ribosomes^52^, which are highly abundant. We collected 68,290 movies and manually picked 53,882 particles for single particle analysis (SPA) cryo-EM (Figure S1A). From these particles, we reconstructed maps of the ATP synthase dimer (11,338 particles), mitochondrial Hsp60 (1,154 particles), and OXPHOS complex III (5,208 particles) (Figure 1C and S1B–C).

Initial *ab initio* reconstruction of all manually picked particles resulted in two classes; one consistent with the size and shape of an ATP synthase dimer (14,917 particles) and another with Hsp60 (7,597 particles) (Figure S1B)^24,28,53–55^. The set of particles consistent with ATP synthase were filtered down to 11,338 particles through three-dimensional (3D) classification resulting in an initial 7–10 Å 3D refined map. Density for one monomer was weaker, likely reflecting flexibility between monomers or damage at the air-water interface (Figure 1C)^53^. Optimization of the image processing workflow—including masking and recentering on individual ATP synthase monomers or the dimer interface within each ATP synthase dimer and performing local refinements—improved maps of the overall ATP synthase monomer (4.2 Å), F_1_ region (3.8 Å), OSCP region (4.3 Å), and transmembrane dimer interface (4.1 Å) (Figure 1D,E, S1B, S2A–D,G–I, and Table 1). A composite map of the ATP synthase monomer was generated by combining the local maps of the F_1_ and OSCP regions, with that of the ATP synthase monomer (Figure 1D). Together with the locally refined dimer interface, these maps reveal the first structure of an ATP synthase from the Amoebozoa eukaryotic superfamily (Figure 1C–E).

For the Hsp60 class, the set of particles was filtered by several rounds of two-dimensional (2D) and 3D classification resulting in a 3.9 Å map of Hsp60 (final set of 1,154 particles) (Figure S1B, and S2E,J and Table 1). Additionally, careful re-examination of all particles in the dataset revealed another class that resembled OXPHOS complex III (Figure S1B, *blue boxed class*)^56^. Subsequent template-based picking approaches followed by several rounds of 2D and 3D classification, and 3D refinement resulted in a 3.9 Å resolution map of a complex III dimer (CIII_2_) from 5,208 particles (Figure S1C, S2F,K and Table 1).

### Integrated approaches to build atomic models

To overcome the limited particle number due to the enrichment- and purification-free nature of our structure determination approach, we utilized sequence agnostic model building by ModelAngelo^57^ and used HMMER^58^ to search the generated Hidden Markov model (HMM) profiles against the *Acanthamoeba* proteome^42^ to verify the identity of the maps we obtained (Figure S4). ModelAngelo built partial peptides into regions of the ATP synthase maps with sufficient resolution (F_1_ domain, OSCP region, and F_o_ dimer region) (Figure 1D,E and S4A–C). All hits were mitochondrial proteins and resulted in the assignment of ATP synthase subunits α, β, γ, OSCP, d, f, 6, b, 8, e, g, and i/j as well as the *Acanthamoeba*-specific ATP synthase subunits – ACANB14707 and mitochondrial MDH (AcMito-MDH) (Figure 1D,E,F and S4A–C)^43,59,60^. The remainder of the subunits were manually built into the map using COOT informed by previously solved structures of homologs or AlphaFold3 predictions^58,59^. While the majority of a dimeric AlphaFold3 model of AcMito-MDH agreed with the density of the map, the N-terminal region differed, as indicated by the ModelAngelo build and was manually rebuilt using COOT (Figure S4A–C)^57,62^.

After subunits identified and placed by ModelAngelo were manually built to completion, there remained unaccounted regions of the map. Previous BN-PAGE complexome data identified additional components, subunits c, δ and ε, IF1, and ACANB04460 (Figure 1F, S3A,B), that co-migrated with ATP synthase subunits^43,44^, which were verified by IP-MS analyses (reported in Stefely et al., co-submitted)^43^. While AlphaFold3 failed to predict the entire structure of ATP synthase, predictions of the rotor (c-ring and subunits δ and ε, and part of the γ subunit) and ACANB04460 with other components of the peripheral stalk resulted in models that agreed with the unassigned density in the cryo-EM map and were manually placed and fitted (Figure S4F). Within the F_1_ domain we observed a helical density between one α/β pair. Based on previously solved structures of eukaryotic ATP synthases and consistent detection by multiple IP-MS-method experiments^43^, we assigned this density to the inhibitory factor, IF1 (Figure S3B)^22,28,63^. The rest of the map where helices were visible, but unassigned, was manually built using COOT (Figure 1D, *gray*).

Model building for the other two structures obtained from the dataset followed more standard workflows. For the mitochondrial Hsp60 map, the mitochondrial Hsp60 (ACANB13112) was the top hit for all peptides built. The rest of the map was slightly disordered, but a few helices in the apical domain could be built using an AlphaFold3^61^ model of the monomer as a guide in COOT^62^ (Figure S1B). This structure closely reflects the structure of the human mitochondrial Hsp60^54,64^. In the CIII_2_ electron density map, ModelAngelo^57^ and HMMER^58^ identified, placed, and built eight CIII subunits *de novo*—UQCRC2, UQCR11, UQCRB, UQCRQ, MPP-β, CYC1 and MT-CYB (Figure S4E and S5A)^57^. The remaining unassigned density agreed with the placement of UQCR10 and UCRH from an AlphaFold3^61^ prediction of the subunits identified from complexome analysis^43,44^. The Rieske subunit was the only subunit identified that could not be built into the cryo-EM map (Figure S5A). While previous studies have identified the composition of complex III in *Acanthamoeba*^44^, this is the first structure confirming a unique dimerization arrangement, a heterotetramer of UQCRC2 and MPP-β (Figure S5A,B)^56,65,66^. Phylogenetic analysis across the tree of life (Chen et al, co-submitted^67^) predicts that an ancestral duplication gave rise to MPP-α and UQCRC2 paralogs in amorpheans and that the MPP-β and UQCRC1 paralogs in opisthokonts may have arisen independently in animals and fungi (Figure S5C). This evolutionary history is consistent with the distinctive subunit composition observed in the AcCIII_2_ structure. Taken together, these observations on *Acanthamoeba* Hsp60 and CIII demonstrate the discovery potential of imaging wild-type mitochondrial lysate.

### Overall structure of the amoebozoan ATP synthase dimer

Due to their prominence in the micrographs, dimers of ATP synthase dominated our particle picks (Figure S1A). The overall AcATP synthase structure reveals a pyramid shaped head or F_1_ region, made of three α and three β subunits surrounding two long helices from the γ subunit. The peripheral stalks of each monomer are offset and the dimer angle between the two monomers is 70° (Figure S6), indicating that the AcATP synthase dimer is a Type IV dimer^3^. We observe a distinct “hat” region with large density sitting above the OSCP subunit that is distinct from previous ATP synthase structures.

### Structure of the catalytic F_1_ domain

Atomic models built by focused local refinement of a single monomer within the dimer reveal a monomeric architecture that resembles ATP synthase structures from other organisms including mammals, fungi, *Trypanosoma*, *Toxoplasma*, and other protists^22,24,28^. All core subunits were built as well as three *Acanthamoeba*-specific subunits in the peripheral stalk—ACANB04460, ACANB14707, and AcMito-MDH. ACANB04460 makes contact with subunits d, b, and 8, suggesting this additional subunit contributes to the stabilization of the peripheral stalk. ACANB14707 and AcMito-MDH sit above OSCP, creating the “hat” noted earlier no obvious interaction with the F_1_ catalytic domain or the F_o_ rotor.

As in other ATP synthases, the F_1_ domain contains three copies each of the α and β subunits, labeled as α_E_/β_E_, α_DP_/β_DP_, and α_TP_/β_TP_ following established nomenclature^22,24,29,63^. The nucleotide occupancy in the F_1_ domain is consistent with an inhibited state. In the cryo-EM map, all nucleotide binding sites are visible (Figure 2A,B). Each of the non-catalytic α subunits nucleotide binding pockets in the are occupied by an ATP molecule while the occupancy of the three catalytic β subunits varies (Figure 2A,B). The nucleotide binding pocket in β_E_ is empty and adopts an open conformation whereas β_DP_ and β_TP_ are both occupied by an ADP molecule and adopt a closed conformation (RMSD value of 1.5 Å, Figure 2C). Density consistent with a Mg^2+^ ion is also observed in the β_TP_ binding site, but typically this pocket is occupied by an ATP molecule. Despite the observation of an ADP molecule in both β_TP_ and β_DP_, there are subtle differences between the two active sites—including a repositioning of two arginines, one in the β subunit (Arg_375_) and one in the neighboring α subunit (Arg_204_) (Figure 2D). α_DP_-Arg_204_ (*gray*) reaches into the binding site, coordinating with the γ-phosphate in the ADP molecule in the β_DP_ active site. β_TP_-Arg_375_ (*gray*) comes down closer to the β_TP_ active site, occupying the position α_DP_-Arg_204_ (*blue*) occupies in the β_DP_ active site (Figure 2D). These differences may underlie phosphate binding and ATP synthesis. The movement of the two arginines may accommodate the binding of a phosphate ion in the β_TP_ active site.

A similar occupancy of the F_1_ domain was observed in four structures of human ATP synthase where it was postulated that these structures reflected an inhibited state or perhaps even represents the reverse activity of the synthase (hydrolysis of ATP to ADP)^63^. In our case, the nucleotide occupancy likely reflects the inhibited state caused by the binding of the inhibitory factor, IF1 (Figure 2E).

In other organisms, IF1 binds to the F_1_ domain via the insertion of a C-terminal helix in the β_DP_ and α_DP_ interface, but the mode of N-terminal binding varies^22,28^. In mammals, the N-terminus forms a short helix that extends into the F_1_ domain interacting with the coiled-coil of the γ subunit, preventing its rotation^22,68^. In contrast, in the algae *Euglena gracilis*, IF1 lacks an N-terminal helix entirely and an N-terminal loop extends in the opposite direction, no longer contacting the γ subunit^28^. The binding mode of AcIF1 more closely reflects that of mammalian IF1. While only the C-terminal helix could be confidently built into the map, an overlay with the mammalian IF1 reveals there is density in our cryo-EM map consistent with the N-terminal helix from SsIF1 (*magenta*), suggesting that AcIF1 (*orange*) binds to the F_1_ domain in a similar manner (Figure 2E).

While AcIF1 binds to the F_1_ domain in a manner similar to mammals, the lack of a pH sensing histidine suggests that although the inhibitory mechanism may be conserved, the mode of regulation is distinct (Figure 2F, *purple box* and S7)^68–70^. This divergence is further supported by O_2_ perturbation profiling by Stefely et al, co-submitted^43^ (Figure 2G and S3C). While IF1 overexpression has been suggested to regulate ATP synthase activity in anoxic mammalian cancer cells^71^, we observe the opposite in *Acanthamoeba*–IF1 levels decrease relative to other ATP synthase subunits under anoxic conditions (<0.1% O_2_). This suggests a different mode of O_2_-dependent regulation of IF1 expression in *Acanthamoeba*.

The structure also sheds light on lineage-specific extensions to core subunits that were apparent from sequence, but whose functional significance remained unclear^44^. In *Acanthamoeba*, the β subunit has a long C-terminal extension (118 residues) that forms two helices that wrap around a neighboring α subunit and, in one case, contacts the peripheral stalk (Figure 3A and S6). The additional contact of one of the β subunit extensions with the peripheral stalk likely reflects a different evolutionary solution to stabilization of the F_1_ domain to generate ATP. This extension, while present in other amoebozoan β subunits (*D. discoideum*: 58 residues), the *Acanthamoeba* extension is by far the longest (Figure S8). In more distant organisms additional subunits sit up against the α subunits at a similar interface, but in a different manner (Figure 3B,C)^17,24,28,29^. In *Trypanosoma brucei* the lineage-specific subunit, p18, shields most of the matrix exposed surface of the α subunit, burying a total of 26 nm^2^.^24^ In comparison, *Acanthamoeba*, the β subunit extension shields a smaller portion, burying only 13 nm^2^ (Figure 3B,C). This highlights two distinct strategies for F_1_ stabilization – subunit accretion in *T. brucei* versus domain extension of an existing core subunit in *Acanthamoeba* and other amoebozoans – underscoring ATP synthase adaptation throughout evolution, potentially reflecting differences in matrix composition or energy demands across species.

### Structure of the membrane embedded F_o_ domain.

Similarly to the F_1_ domain, most of the F_o_ subunits and the proton-driven mechanism are conserved. Protons enter through the pore formed at the interface between subunit 6 and the c-ring where each c subunit binds to a proton via a conserved glutamate (Glu_53_ in humans and Glu_63_ in *Acanthamoeba*) (Figure S9A)^13,72,73^. The cryo-EM structure reveals the *Acanthamoeba* c-ring stoichiometry (c_9_) differs from yeast (c_10_), mammals (c_8_), and *Trypanosoma* (c_10_)^12–14,24,68,74^. The stoichiometry of the c-ring is indicative of the efficiency of the synthase, i.e. the number of protons translocated across the membrane for every three ATP molecules generated. In *Acanthamoeba*, we hypothesize that ATP synthase transports three H^+^ for every ATP molecule, whereas in mammals the ratio is 2.7 H^+^/ATP^3,12^. Several inhibitory drugs, including bedaquiline (BDQ), block proton transport targeting the conserved glutamate in subunit c^72,73^. While the glutamate is conserved in *Acanthamoeba*, the surrounding residues fail to create the negative binding pocket necessary for BDQ binding, suggesting BDQ would be ineffective against *Acanthamoeba* ATP synthase (Figure 9B)^72,73,75^.

Inspection of transmembrane proteins flanking the c-rotor reveals a different packing of the transmembrane segments (TMS) compared to previously solved structures^24^. Similar to mammalian subunit b, the *Acanthamoeba* subunit b traverses the membrane twice via an N-terminal helix (b_H1_) and a long helix (b_H2_) that helps form the peripheral stalk (Figure 4A, *mint green*), but the positioning of this helix differs. In *Acanthamoeba* ATP synthase, b_H1_ packs closer to b_H2_ compared to that in pig ATP synthase, by 12.9 Å, which allows for subunits g and e to pack closer to the c-ring than in mammals (Figure 4A,B). In *Trypanosoma* ATP synthase, while subunit b possesses a single transmembrane segment (TMS), an additional subunit ATPEG4 sits between the TMSs of subunit b and f (Figure 4A, *yellow/green*). This organization of the helices in *T. brucei* ATP synthase, results in a similar TMS packing to that of mammals. In *Acanthamoeba*, the TMS of all core subunits pack tightly together, resulting in a compact ATP synthase monomer in the transmembrane region that is also reflected in the dimerization interface.

### *Acanthamoeba*-specific dimer interface

The tight packing of the TMS within the monomeric F_o_ domain is reflected in the dimer interface. Mediated by transmembrane subunits e and g, AcATP synthase dimers form a conserved ancestral interface, which has also been observed in *Trypanosoma* and mammalian ATP synthases (Figure 4C)^24^. In *Acanthamoeba*, the ATP synthase dimer interface is predominantly mediated by subunit e, which forms a central ‘x’, while subunit g flanks the interface, running parallel to the membrane surface and then inserts itself between the matrix facing side of the ‘x’ (Figure 4D).

Strikingly, the conserved GXXXG motif present in subunits e and g in other species that allow for close interactions between their TMSs is absent in *Acanthamoeba* subunit g (Figure 4D, *orange*)^24^. Instead, the contact between each subunit e is mediated by hydrophobic interactions, resulting in a tighter interaction between the two in *Acanthamoeba* (Figure 4D). The absence of close contacts between subunits e and g via the GXXXG motifs demonstrates that this sequence is not sufficient to predict this ancestral dimerization interface, but rather one of several binding modes for achieving oligomerization. This alternative binding mode of the e subunits may be related to the unusual origin of subunit e in *Acanthamoeba*, which we predict is the product of a recent horizontal gene transfer from prokaryotes that replaced the canonical e subunit found in opisthokonts (Figure S10). Using protein orthogroups defined as part of our MitoTOL project (details in Chen et al., co-submitted^67^), we observe only one evolutionary distant eukaryotic protein homolog from *Caenorhabditis elegans* within this orthogroup. A HMMER search of the protein members of this orthogroup reveals a link to FtsL—a single transmembrane helix found in the outer membrane of bacterial cells^58,76^. The tighter hydrophobic interface and packing of the transmembrane segments in the F_o_ and peripheral stalk domains of *Acanthamoeba* may provide increased stability, potentially compensating for the absence of auxiliary dimer-stabilizing proteins found in other systems like *Trypanosomes* or algae^24,28,29^.

Since IMM structure is tightly linked to ATP synthase dimer packing, these findings reveal a minimal dimeric unit for supporting membrane curvature. Future work will be required to fully understand how the tighter hydrophobic interface in *Acanthamoeba* visualized in this structure ultimately contribute to cristae morphology^41^.

### Extensions of core subunits compensate for missing subunits

Most core ATP synthase subunits were identified and placed within the maps, with the exception of subunits k and h. Notably, no homolog for subunit k has been identified by sequence-based searches^60^ nor experimental methods (IP-MS^43^ and BN-PAGE complexomics^43,44^) and there is no density consistent with an additional transmembrane subunit in the cryo-EM reconstruction. Together, this suggest that *Acanthamoeba* lacks a subunit k and no other subunit compensates for its absence.

Similarly, no homology for subunit h has been identified by sequence-based^60^ or experimental approaches^43,44^. In other organisms, subunit h is integrated into the peripheral stalk, residing in the matrix, furthest from the membrane and closest to OSCP. While we observe helical density in this region, we are unable to assign this density and built them as poly-alanine helices (Figure 1D, *gray helices*). Two candidates could account for those helices–OSCP and subunit d (499 vs 161 residues in humans) are considerably longer than in other organisms. AcOSCP possesses C-terminal extension could fold into helices to connect to the rest of the stalk (Figure S11). Perhaps in *Acanthamoeba*, these subunits (OSCP & d) evolved to have additional residues to compensate for a missing subunit, reminiscent of the subunit b and h fusion in *Plasmodium* and other related organisms^60^.

### Structural characterization of a malate dehydrogenase integrated within the AcATP synthase stalk

Local refinement of the prominent density above OSCP in the AcATP synthase resulted in a map that revealed two *Acanthamoeba*-specific ATP synthase subunits—AcMito-MDH and ACANB14707. Two copies of the same AcMito-MDH helix were identified directly from the refined map, indicating that a AcMito-MDH dimer sits atop OSCP in each ATP synthase monomer (Figure 5A). Compared to other MDHs, both cytosolic and mitochondrial, AcMito-MDH has a 76 residue N-terminal extension (Figure 5B, *gray* and S12)^59^. This extension accounts for the entirety of six helices, three from each AcMito-MDH monomer (outlined in *gray*) that wrap around a short 25 amino acid helix formed by ACANB14707 (*hot pink*), like a pair of hands wrapping around a baton (Figure 5A). In this configuration, ACANB14707 acts as a scaffold for AcMito-MDH to attach to ATP synthase, so we here on out refer to it as MDH-ATP synthase adaptor (MASA) subunit. In this configuration the predicted active site remains accessible.

Both we and others^44^ observed in BN-PAGE complexomics that AcMito-MDH migrates at the same molecular weight as other members of ATP synthase—suggesting robust association between the two (Figure 5C and S3B). However, whether this was simply co-migration of AcMito-MDH and ATP synthase has been a question, as MDH is not typically thought to interact with ATP synthase^44^. This structure verifies that a MDH dimer is indeed incorporated into AcATP synthase and reveals how the enzyme is physically anchored via interactions with the MASA subunit and OSCP. To understand environmental stressors that could contribute to this assembly, we explored perturbational profiling analysis by Stefely et al, co-submitted^43^. Under most perturbations (O_2_ levels and carbohydrate and iron sources), AcMito-MDH displays a similar proteomic profile and tracks with other ATP synthase subunits (Figure 5D and S3C). The only instance the proteomic profile differs is under iron starvation, implying the integration of AcMito-MDH into ATP synthase is stable and not regulated by carbohydrate sources.

To identify any potential conformational changes in AcMito-MDH induced upon incorporation into ATP synthase via the N-terminal interaction with the MASA subunit, we solved a 2.3 Å crystal structure of apo AcMito-MDH (Figure 5E and S13A–C). The crystal structure revealed the same dimeric conformation as the cryo-EM structure (Root Mean Square Deviation (RMSD) of 0.56, Figure 5A,E, and S13E), but with an unresolved N-terminus. Collectively, the crystal and cryo-EM structures reveal that integration of AcMito-MDH into ATP synthase does not drastically change the dinucleotide binding pocket of the TCA cycle enzyme.

### Catalytic activity of AcMito-MDH is impacted by integration into ATP synthase

Next, we sought to determine if this newly identified AcMito-MDH functions as a conventional MDH, by recombinantly expressing and purifying AcMito-MDH alone and measuring its steady-state kinetics (Figure 5F, *gray* and S13A,B)^77,78^. MDH catalyzes the conversion of NAD^+^ and malate to NADH and oxaloacetate in the TCA cycle, but is also capable of the reverse reaction, catalyzing the conversion of NADH and oxaloacetate into NAD^+^ and malate^45,46^. As the forward reaction is energetically unfavorable at neutral pH, the reverse reaction is typically measured *in* vitro^78^. We measured the change in absorbance at 340 nm reflecting the conversion of NADH to NAD^+^ with varying amounts of oxaloacetate and 1 nM of protein (Figure 5F)^77,78^. AcMito-MDH converted oxaloacetate to malate with a catalytic efficiency of 7.43 × 10^6^ M^−1^s^−1^, which is similar to measured k_cat_/K_M_ values of other mitochondrial MDHs (8.85 × 10^6^ M^−1^s^−1^ in *C. elegans*^78^ and 8.1 × 10^4^ M^−1^s^−1^ in *T. emersonii*^79^) (Figure S13F).

Since the active site of AcMito-MDH is still accessible in the cryo-EM structure, we sought to investigate if integration into ATP synthase impacted the enzymatic activity of AcMito-MDH. We isolated the AcATP synthase complex via immunoprecipitation (IP) of a C-terminally HA-tagged AcMito-MDH followed by cleavage at a 3C site (Figure S13G,H) and then tested the enzymatic activity of AcMito-MDH in this purified complex (Figure 5F). The purified sample retains the ability to use NADH to reduce oxaloacetate to malate, confirming that AcMito-MDH remains catalytically active in the complex. Mutation of a conserved histidine involved in proton transfer, His_294_, to an alanine produced a catalytically dead MDH enzyme (Figure 5F, *blue line* and S13G). Compared to recombinantly purified AcMito-MDH alone, the purified AcATP synthase shows an ~85% reduction in K_M_, suggesting that integration into ATP synthase increases AcMito-MDH substrate affinity (Figure 5G and S13F). We also observe an increase in K_i_ indicating a higher substrate concentration is required before inhibition of AcMito-MDH occurs (Figure 5H). These results suggest that integration of AcMito-MDH within AcATP synthase expands the working substrate concentration range of the MDH, potentially conferring metabolic flexibility to mitochondrial flux.

As our structure reveals the N-terminal extension in AcMito-MDH interacts with the MASA subunit, we investigated whether this extension contributes to the observed changes in substrate binding affinity and inhibition of AcMito-MDH (Figure 5A). We generated an *Acanthamoeba* strain that expressed an HA-tagged AcMito-MDH lacking the N-terminal extension and purified an AcATP synthase_MDHΔNTD_ complex via IP and elution through HRV 3C cleavage (Figure S13G,H). Removal of the three N-terminal helices increases K_M_ to levels comparable to that of the AcMito-MDH alone, suggesting that the extension is key to the enhanced substrate affinity observed in AcMito-MDH when integrated into AcATP synthase (Figure 5F–H, S13F). In the structure, the MDH-NTD-MASA interaction is on the opposite side of the active site, a region found to be important for allosteric MDH regulation in other organisms^80,81^. This is consistent with the structural observation of these helices wrapping around the MASA subunit, indicating a role for this interaction in the allosteric modulation of AcATP synthase:MDH activity.

To investigate if ATP synthase impacts AcMito-MDH activity through this structural integration, we measured AcMito-MDH activity of the complex in the presence of different ATP synthase substrates, ATP and ADP (Figure 5I). We found that two nucleotides impact the working substrate concentration range and catalytic activity of AcMito-MDH differently. The addition of ATP decreases the catalytic rate of AcMito-MDH (k_cat_), while the addition of ADP increases k_cat_ (Figure 5J and S13F). Furthermore, the addition of ADP increased the K_M_ of AcMito-MDH, shifting substrate affinity to levels comparable to recombinantly purified AcMito-MDH alone. Our enzymatic studies confirm that an active MDH is integrated into AcATP synthase and indicate that MDH activity is allosterically modulated by ATP synthase substrates, revealing not only a physical link between OXPHOS and the TCA cycle, but also suggesting regulatory mechanism.

## DISCUSSION

In this study, we present the first high-resolution structure of an ATP synthase from the Amoebozoa eukaryotic supergroup, an important evolutionary “cousin” outgroup to Opisthokonta (the supergroup that includes humans and yeast). By integrating cryo-EM, proteomics, and computational modeling, we characterized lineage-specific subunits and extensions to conserved subunits in *A. castellanii* ATP synthase. A notable *Acanthamoeba*-specific feature was an MDH dimer integrated into each ATP synthase monomer, revealing a physical connection between OXPHOS and the TCA cycle (Figure 6). Together these findings fill an important phylogenetic gap in understanding mitochondrial ATP synthase structure and evolution.

The combination of cryo-EM, unbiased model building, and proteomics was critical for building an atomic model of ATP synthase. The overall architecture of AcATP synthase is strikingly similar to that of other eukaryotes, with a conserved F_1_ catalytic head, F_o_ proton channel, and connecting stalks^11,17,22,24,26,28,29,63,68^. The well-resolved AcATP synthase F_1_ domain revealed an inhibited state resembling the inhibited human ATP synthase. Yet despite this shared core, our analyses reveal numerous *Acanthamoeba*-specific features. Three *Acanthamoeba*-specific subunits were identified and placed into the peripheral stalk: ACANB04460, MASA subunit, and AcMito-MDH. The identification and placement of multiple novel *Acanthamoeba*-specific proteins in AcATP synthase directly from the electron density map emphasizes the advantages of structure determination from native sources and highlights the structural diversity of OXPHOS complexes in eukaryotes.

A recent report also identified a weak link between MDH and ATP synthase in mammals that could only be detected by cross-linking MS^82^, highlighting the broader relevance of this link and the need for a tractable system to investigate its potential regulatory roles and biological relevance. The *Acanthamoeba* ATP synthase-MDH complex is an ideal system for such studies as it is a stable assembly that can be purified and manipulated through mutations to individual subunits. The cryo-EM structure reveals that the ATP synthase:MDH interaction in *Acanthamoeba* is facilitated by both a lineage-specific scaffolding subunit and three additional N-terminal helices in each AcMito-MDH monomer. These *Acanthamoeba*-specific adaptations mediate the stable assembly of two enzymes from two distinct metabolic pathways–OXPHOS and the TCA cycle (Figure 6). Here we lay the foundation for future work by purifying the complex from *Acanthamoeba* and demonstrating that AcMito-MDH remains active within it, and that ATP synthase substrates can differentially modulate MDH activity.

*Acanthamoeba* mitochondria show unusual features of malate metabolism. The glyoxylate shunt, an anabolic pathway that bypasses many TCA enzymes converting acetyl-CoA to succinate for carbohydrate synthesis^83^, is mitochondrially localized in *Acanthamoeba*, rather than in specialized peroxisomes (fungal and plants) or the cytosol (bacterial)^43,59^. Interestingly, its two core enzymes, isocitrate lyase and malate synthase are fused as a single polypeptide (MS-ICL). This raises the possibility that in the amoebozoan, the regulatory effects we observe in MDH through integration into ATP synthase impact the glyoxylate shunt as well or instead of the TCA cycle. Future work investigating the MDH:ATP synthase interaction under different carbon sources will help clarify this. Nevertheless, the co-occurrence of an MDH integrated into an ATP synthase in *Acanthamoeba* alongside a mitochondrial glyoxylate shunt (Figure 6)^43,59^ underscores the primacy of malate metabolism in *Acanthamoeba* mitochondria.

In addition to lineage-specific subunits, we also visualize key features of core subunits. In amoebozoans there is a C-terminal extension on the β subunit that folds into two helices and wraps around a neighboring α subunit. In some other Type IV ATP synthases this outcome is achieved through subunit accretion instead of a modification to a core subunit^24,84^. The C-terminal extensions of one of the β subunits interfaces with the peripheral stalk resulting in a distinct mode of F_1_ stabilization not observed in other organisms like mammals and *Polytomella*—where the N-terminus of an α subunit integrates into the peripheral stalk^11,17,22,29,63,68^. Together, these features highlight how extension of a core subunit can serve as an alternative evolutionary solution to shielding the α subunit and stabilizing the F_1_ region.

The *Acanthamoeba* ATP synthase dimer retains an ancestral dimerization interface while exhibiting a different mode of dimerization. Rather than the possessing GXXXG motifs in the e and g subunits that facilitate TMS packing in other organisms, a hydrophobic interface on the lumen facing side of the membrane drives dimerization in *Acanthamoeba*. This interface results in a tighter packing between two ATP synthase monomers and demonstrates how alternative structural solutions can achieve a similar dimerization interfaces^22,24^. The observation of the ancestral dimerization interface in AcATP synthase, together with phylogenetic evidence that amoebozoans are a closer neighbor to opisthokonts than more diverged protists (*Trypanosoma*, *Polytomella*, *Euglena*)^32,34,85–87^, support the notion that the canonical dimer observed in mammals and fungi is Opisthokonta-specific.

Our integrative approach demonstrates the power of combining cryo-EM on native mixtures, proteomics, and computational tools to characterize architectural specialization in protein complexes from a diverged protozoan pathogen. Similar strategies may be used to expand the structural understanding across the eukaryotic tree of life, ultimately structurally linking molecular evolution to eukaryotic diversity.

### Limitations of the study

Our structure provides the first amoebozoan ATP synthase structure, identifying and characterizing clade specific adaptations. While it is a technical feat to solve the structure from a small number of starting particles (~15,000), the limited particle count from our dataset constrained us to only capturing the inhibited IF1 bound state. As no other structures of *Acanthamoeba* ATP synthase exist and prediction algorithms fail to predict the entire complex, we have no active states available for comparison. Future studies of samples enriched for ATP synthase could capture these active states. Furthermore, we solubilized mitochondria lysate using detergent, which could have biased oligomerization to the dimer state and may not reflect the distribution of oligomers *in situ*. In addition, our *in vitro* enzymatic characterization of AcMito-MDH confirmed activity in isolation and in complex with ATP synthase. Future work within the organism or with isolated mitochondria can measure ATP synthesis in the presence of different metabolites to provide insight on the regulatory role of the MDH on ATP synthase.

## RESOURCE AVAILABILITY

### Lead contact

Further information and resources, strains, or data will be available upon request. Requests should be directed to the lead contact, Luke Chao (chao@molbio.mgh.harvard.edu).

### Materials availability

All data and plasmids will be available upon request.

### Data and code availability

Cryo-EM maps have been deposited in the Electron Microscopy Data Bank (mitochondrial Hsp60: EMD-74149, complex III: EMD-74150, ATP synthase dimer: EMD-74151, composite map of ATP synthase monomer: EMD-74152, ATP synthase monomer: EMD-74153, ATP synthase F_1_: EMD-74155, ATP synthase MDH-OSCP: EMD-74156, and ATP synthase F_o_ dimer: EMD-74154). Atomic coordinates built into the Hsp60, complex III, ATP synthase monomer, ATP synthase F_1_ ATPase, ATP synthase MDH-OSCP, and ATP synthase F_o_ dimer cryo-EM maps have been deposited in the Protein Data Bank (PDB) under the accession codes 9ZFR (Hsp60), 9ZFS (complex III), 9ZFT (ATP synthase monomer), 9ZFV (ATP synthase F_1_ ATPase), 9ZFW (ATP synthase MDH-OSCP), and 9ZFU (ATP synthase F_o_ dimer). The crystal structure of AcMito-MDH was also deposited to the PDB under the accession code: 9ZFX.

## STAR METHODS

### EXPERIMENTAL MODELS

#### Cell culture

*Acanthamoeba castellanii* strain Neff (wild type) was cultured in vented tissue culture flasks (182-cm side-arm flasks) with standard medium (SM) (20 g/L proteose peptone, 1 g/L yeast extract, 4 mM MgSO_4_, 0.4 mM CaCl_2_, 3.4 mM sodium citrate, 50 uM Fe(NH_4_)_2_(SO_4_)_2_, 2.5 mM Na_2_HPO_4_, 2.5 mM KH_2_PO_4_, pH 6.5, 100 mM glucose, Pen-Strep) at 30 °C in a stationary dry incubator.

### METHOD DETAILS

#### Mitochondrial isolation and sample preparation in *Acanthamoeba castellanii*

Crude mitochondria were isolated by differential centrifugation as essentially detailed separately (Stefely et al, AcMitoCarta, co-submission). Briefly, *Acanthamoeba castellanii* strain Neff, wild type, was cultured in standard media until the cells reached near-confluency (90–95% confluent, adherent cells). Cells were harvested via centrifugation (800 *g*, 5 min, 4 °C) and resuspended in phosphate buffered saline (PBS) with 0.5 mM PMSF (50 mL, 4 °C) and then centrifuged (800 *g*, 5 min, 4 °C) again. Cell pellets were resuspended in sucrose-HEPES-EDTA buffer with BSA and 2x protease inhibitors (SHEB2xP) (0.28 M sucrose, 10 mM HEPES-KOH, pH 7.2, 0.2 mM EDTA, 1 mg/mL fatty acid free BSA, 2x Roche complete protease inhibitor mix, 0.5 mM freshly added PMSF) (final volume to 30 mL, 4 °C) and homogenized with a Dounce homogenizer (15 mL of cell suspension in a 15-mL glass Dounce homogenizer, 15 strokes with loose pestle, 10 strokes with tight pestle). The whole-cell homogenate was centrifuged at slow speed (800 *g*, 10 min, 4 °C) twice to pellet intact cells and nuclei. The slow-spin supernatant was recovered, transferred to a clean 50-mL conical tube, and centrifuged at high speed (8,000 *g*, 10 min, 4 °C) to pellet mitochondria. The mitochondrial pellet was resuspended into SHEBP (4 °C), aliquoted into microcentrifuge tubes, and centrifuged at high speed (10,000 *g*, 10 min, 4 °C). The isolated mitochondrial pellets were frozen in liquid nitrogen and stored at −80 °C.

Isolated mitochondria with a total of approximately 450 μg protein concentration were lysed in 200 μL of lysis buffer (50 mM HEPES pH 7.9, 300 mM NaCl, 4 mM MgCl_2_, 20 μM CaCl_2_, 0.1 mM Dithiothreitol (DTT), and 0.5% n-dodecyl-β-D-maltoside (DDM)) by gentle pipetting and then left of ice for 10 minutes. The lysate was centrifuged at 14,000 *g* for 10 min to separate the soluble fraction from the membrane fraction.

#### Cryo-EM grid preparation and data collection

EM samples were collected from the soluble fraction and filtered with a 0.1 μm Durapore^®^ PVDF filter. Immediately after filtering, 3 μL at a concentration of 0.8 mg/mL was deposited onto Holey carbon grids (Quantifoil Cu-300 Mesh R1.2/1.3) that were glow discharged under air with 15 A plasma current for 90 seconds with a Pelco easiGlow, Emeritech K100X. In an FEI Vitrobot Mark v4 x2 chamber set to 4 °C and 100% humidity, grids were blotted for 3–4 s at a force of 15 and frozen by plunging into liquid ethane. Data were collected on four separate occasions using an automated data collection program, EPU (TFS), on an FEI Titan Krios equipped with an energy filter (20 eV slit width) at 300 keV and a Gatan K3 direct detector. Beam illumination was adjusted to a fluence of 12.4–17.8 e-/pixels/second. Images were collected using a defocus range of −0.6 to −2.0 μm with a step size of 0.2 using super resolution mode at a calibrated pixel size of 0.411 Å/pixel. Using counting mode, 2.01–2.52 s images were collected with a frame rate of 42–50 ms and a dosage of 0.92–1.06 e-/Å per frame. Before data transfer, collected movies were downsampled to a corrected pixel size of 0.822 Å/pixel.

#### Image processing

A total of 68,290 movies were initially processed using cryoSPARCv4.7.0^88^ to generate aligned dose-weighted micrographs (Figure S1A). The aligned micrographs underwent contrast transfer function refinement. 59,894 particles were manually picked and extracted at 4x bin and then six *ab initio* models were generated using cryoSPARC.

##### ATP synthase

One class was consistent with the expected shape and size of ATP synthase (a total of 14,917 particles). Particles were further filtered via an additional round of *ab initio* model generation by cryosparc resulting in a class consistent with the expected shape of ATP synthase consisting of 11,338 particles. A mask of a single monomer of the ATP synthase dimer was generated using relion_mask_create^89^ and particle subtraction was carried out to isolate the single masked monomer. These subtracted particles were then subjected to a round of homogeneous refinement. Next particles were centered on the mask of the ATP synthase monomer, refined and then re-extracted at 1x bin. Both monomers of each ATP synthase dimer complex used for the original reconstruction were selected and picks underwent a round of *ab initio* model generation. Two classes were consistent with the shape of an ATP synthase monomer (a total of 14,352 particles). A round of 3D heterogeneous refinement was carried out to produce a highly filtered class of 10,867 particles. These particles were refined to 4.2 Å.

The map was then subjected to local refinement of different domains, namely the F_1_ domain, the peripheral stalk, and the MDH-OSCP regions (Figure S1B). These local refinement jobs were performed with soft masks of 6 pixels extended by 4 pixels resulting maps with estimated resolutions of 3.77 Å, 5.5 Å, and 4.3 Å, respectively (Figure S1B and S2A,B,C). Local refinement of the c-ring and gamma stalk did not improve resolution.

Similarly to the ATP synthase monomer, the transmembrane dimer interface was resolved by centering the original 14,194 particles on a mask of the transmembrane region and re-extracted at 2x bin (Figure S1B). These particles underwent a round of 2D classification for further filtering resulting in 11,768 particles. These particles were refined to 4.4 Å. Subsequently C2 symmetry was applied resulting in a refinement that reached 4.23 Å (Figure S1B and S2D). The map when the subjected to local refinement with a soft mask of 6 pixels extended by 4 pixels.

##### Hsp60

One class was consistent with the size and shape of mitochondrial Hsp60 consisting of 7,597 particles that resembled Hsp60 (Figure B). two-dimensional (2D) classes were generated from the particles that did not fall into the initial ATP synthase dimer *ab initio* class and used as templates to pick more particles. These 1,620,633 picks were then subjected to several rounds of 2D classification resulting in a final set of 25,122 particles (Figure S1B). These particles then underwent a round of *ab initio* reconstruction. Two classes stood out, one consistent with Hsp60 (1,154 particles) and the other with complex III (3,533 particles). Taking the particles that fell into the Hsp60 class, we performed homogeneous refinement and applied D7 symmetry, resulting in a map with a resolution of 3.8 Å (Figure S1B and S2E).

##### Complex III

2D classes were generated from a structure of bovine complex I and used for template picking (Figure S1C). Template picking resulted in 1,374,612 picks. These particles were filtered to 63,000 particles via several rounds of 2D classification. *Ab initio* three-dimensional (3D) models were generated using these particles and a class with 5,794 particles was consistent in size and shape to Complex III. These particles then went through one round of heterogenous refinement resulting in a set of 5,208 particles. This final particle set was refined and C2 symmetry was applied, resulting in a 3.9 Å resolution map (Figure S1C and S2F).

#### Model building

Structure prediction by AlphaFold3^61^ failed for a complete model of ATP synthase including all known and identified by mass-spectrometry subunits. Breaking the subunits into sections and predicting a F_o_ and F_1_ region independently also failed. Model building was done in sections, the F_1_ domains, the AcMito-MDH-OSCP region, the transmembrane dimer interface, and the peripheral stalk (Figure S4A,B,C). Within the F_1_ domain, the α (residues 29–125, 26–126, 25–49, 61–78, 82–128, 131–173, 143–162, 150–163, 177–292, 177–199, 178–237, 202–264, 238–323, 266–321, 295–318, 322–407, 327–401, 329–408, 414–494, 414–497, 413–490, 491–517, and 499–519), β (residues 72–193, 73–115, 74–97, 100–217, 119–288, 195–239, 245–273, 256–435, 290–381, 296–466, 323–353, 384–407, 424–460, 440–466, 470–501, 474–497, 475–550, 507–523, 531–552, 582–650, and 597–624), and γ (residues 44–82 and 262–308) subunits were identified using ModelAngelo searching against the entire *Acanthamoeba* proteome (Figure S4A)^57^. Using the same method for the AcMito-MDH-OSCP region, AcMito-MDH (residues 68–108, 210–327, 311–364, and 391–420), OSCP (197–221 and 257–274), and the MASA subunit (63–87) were identified and placed in the AcMito-MDH-OSCP map (Figure S4B). Within the transmembrane dimer interface, ModelAngelo identified and placed subunits 6 (residues 30–48, 56–83, 60–79, 113–134, 159–185, 163–183, 187–236, and 187–237), d (residues 446–467 and 436–468), 8 (residues 13–31 and 10–42), b (residues 9–58 and 11–84), f (residues 56–86 and 55–87), i/j (residues 15–48), g (residues 69–92) and e (residues 41–61, 40–56) from the entire *Acanthamoeba* proteome (Figure S4C). There was an additional transmembrane helix unaccounted for after *de novo* building. The peripheral stalk region of the ATP synthase monomer map was built based on the placement from the transmembrane dimer interface map. Subunits 6, d, 8, f, i/j, and b were extended to fit the rest of the density based on AlphaFold3 predictions. Based on AlphaFold3 predictions, the rotor (c-ring, δ, and ε) and ACANB04460 were assigned to additional density remained unaccounted for (Figure S4F).

After placement, all models were generated using AlphaFold3^61^ and rigid body fitted into the map using chimera^90^. Manual model building was conducted in COOT^62^ and the final model was ran through phenix.real_space_refinement^91^. Areas that could not be unambiguously assigned were not built. Model statistics were evaluated by MolProbity^92^.

For Hsp60, ModelAngelo^57^ was used to build peptides into the final map. These peptides were identified to be ACANB13112 or mitochondrial Hsp60 and covered a signification portion of the protein (residues: 29–206, 366–380, and 412–553) (Figure 4D).

The same approach was taken to build directly into the final complex III map. ModelAngelo^57^ built in peptides from MPP-β (ACANB00313, residues 40–162, 40–75, 77–163, 165–210, 165–243, 215–302, 244–284, 286–301, 305–321, 306–320, 331–360, 346–368, 369–426, 370–426, 428–461, 428–468, and 474–486), CRC2 (ACANB02868, residues 59–130, 72–286, 135–293, 316–333, 316–333, 335–358, 335–376, 362–411, 392–486, and 412–485), UQCRQ (ACANB04471 residues 2–14 and 3–15), Cyc1 (ACANB07833 residues 259–305 and 260–305), UQCR11 (ACANB09854 residues 23–40), UQCRB (ACANB09944 residues 27–75, 30–75, 77–120, and 80–121), and MT-CyB (ACANB15918 residues 20–47, 21–46, 76–106, 77–106, 176–221, 181–221, 224–245, 224–245, 298–344, 302–339, 352–383 and 353–384) (Figure 4E). Two copies of each subunit were built into the map consistent with a dimeric version of CIII_2_. The remainder of the subunits identified by complexomics in Stefely et al., co-submitted and Gawryluk et al., 2012^43,44^, were built using AlphaFold3 predictions^61^.

#### Recombinant protein expression and purification

AcMito-MDH lacking the predicted MTS (residues 36–430) was cloned into a pET28b vector with an N-terminal His-tag and SUMO fusion protein. AcMito-MDH was expressed in *E. coli* NiCo21(DE3) cells (New England BioLabs) in terrific broth (TB) broth at 37 °C and induced by 1mM isopropyl-β- d-thiogalactoside (IPTG) at an optical density (OD600) of around 0.5. After induction, cells were grown at 18 °C for 16 hours and then harvested. Cells were disrupted in lysis buffer (50 mM HEPES pH 7.9, 300 mM NaCl, 10 mM imidazole and 10 mM *β* ME) supplemented with 1 mM phenylmethylsufonyl fluoride (PMSF) and 1 mM benzamidine using a EmulsiFelx-C50 (Avestin). Lysate was separated into the soluble and membrane fractions by centrifugation. The soluble fraction was batch incubated with NiNTA resin at 4 °C for 1.5 hrs. Resin was transferred to a gravity column and washed with lysis buffer and protein was eluted in 50 mM Tris pH 7.5, 300 mM NaCl, 300 mM imidazole, and 10 mM β-ME. Removal of the His-tag was conducted by incubating the elution with ULP1 overnight in dialysis buffer (20 mM Tris, 150 mM NaCl, and 10 mM *β* ME). The dialyzed fraction was ran over a nickel column and washed with lysis buffer to remove the His-SUMO particles and the tag-free *Ac*mito-MDH were collected in the flowthrough and wash. This fraction was further purified using size exclusion chromatography (SEC) through a Superose 6 increase 10/300 (GE) in 20 mM HEPES, 150 mM NaCl, and 10 mM β-ME, pH 7.9.

#### Protein crystallization

Purified AcMito-MDH was concentrated to 12 mg/mL and crystal trays were set using the hanging-drop vapor-diffusion method by equilibrating equal volumes of proteins and well solution (100 mM Tris pH 8, 25 mM MgCl_2_ 23% PEG 3500) in VDX plates with sealant (Hampton Research). Crystals form at 25 °C and were cryoprotected by incubation with 30 μL of well solution with increasing amounts of glycerol (10%, 15%, and 20%) for <5 min before flash freezing in liquid nitrogen.

#### Crystallography data collection

Data was collected at beamline 17-ID-2 FMX at NSLS-II at 13 keV using a custom automated mode. The data set was processed with autoPROC v1.0.5^93–95^ and integrated and scaled using XDS^96–98^ to a resolution of 2.3 Å. Phases were determined using molecular replacement in PHENIX^99,100^ using the model of AcMito-MDH built into the cryo-EM map minus the N-terminal extension. Data was refined with phenix_refine^101^ and MolProbity^92^ was used to identify outliers. Manual building was done in COOT^60^ with the final refinement done in phenix_refine^99,101^. The asymmetric unit contained four copies of AcMito-MDH, two formed a dimer and two formed dimers with a symmetry mate, with residues 108–443 modeled (323 out of 454). The final model has an R/R_free_ of 21.0/25.9 with no Ramachandran outliers. Full refinement statistics are in Figure S13C.

#### MDH enzyme kinetics

Oxaloacetate and NADH stocks were prepared fresh in assay buffer. Initial rates for the conversion of oxaloacetate to malate or malate to oxaloacetate was monitored on a BioTek Cytation5 imaging reader (Agilent) by following the change in absorbance at 340 nm associated with the conversion of NADH to NAD^+^ or NAD^+^ to NADH. Kinetic assays were performed at 25 °C in assay buffer (50 mM Tris pH 7.8, 50 mM KCl, and 5 mM β-ME) with variable oxaloacetate concentrations (from 0 μM to 10 mM), a constant NADH or NAD^+^ concentration (200 nM) and an enzyme concentration of 1 nM for recombinant AcMito-MDH and endogenous ATP synthase_MDH-H294A_ or a calculated concentration of 0.25 nM of ATP synthase-MDH and ATP synthase_MDHΔNTD_. The initial rates were plotted against substrate concentration and fit using Prism to a substrate inhibition equation, v/[E]t = k_cat_ [S]/(K_M_ + [S] + [S]2/K_i_).

#### Endogenous protein expression and purification for enzymatic studies

DNA constructs were cloned into the ectopic circular DNA plasmid GAPDH-EGFP-polyAtailRev vector (from John Samuelson, Boston University). Constructs were amplified via PCR using Q5 Hotstart Master Mix 2x (New England BioLabs, Cat. #M0494S) and PCR fragments were assembled with NEBuilder HiFi DNA Assembly Master Mix (New England Biolabs, Cat. #E2621) or with NEBuilder Q5 Site-Directed Mutagenesis KLD Enzyme Mix (New England BioLabs, Cat. #M0554S). The assembled plasmids containing wild-type AcMito-MDH under the *NDUFS4* promoter and wild-type AcMito-MDH and mutants (AcMito-MDH_H294A_ and AcMito-MDH_ΔNTD_ (deleted residues #34–106)) under the AcMito-MDH promoter followed by a HRV 3C protease cleavage site and subsequent 6xHis- and HA-tags were transformed into chemically competent 5-alpha E. coli (NEB Cat. #C2987H) for scale-up and preparation. Isolated plasmids were sequenced via whole plasmid sequencing.

*A. castellanii* strains were transfected as detailed in Stefely et al. (co-submitted)^43^. Briefly, wild type *A. castellanii* was grown to early log phase (confluency 10–50%). Cells were harvested by centrifugation (1,000 x g, 5 min 4 °C) and washed in Encystation Medium (EM) (20 mM Tris-HCl, pH 8.8, 100 mM KCl, 8 mM MgSO4, 0.4 mM CaCl2, 1 mM NaHCO3) with antibiotics (Pen-Strep). The washed cell pellet was resuspended in EM and manually counted by hemocytometer. A 25-cm side arm culture flask was seeded with 5×10^5^ cells in 2mL of EM and incubated to allow the cells to adhere (30 min, 30 °C). 4ug of plasmid DNA was heated for 5 min at 85 °C and diluted with EM to a final volume of 117 uL, and then 8uL of P3000 reagent was added. Lipofectamine 3000 was diluted in EM and then mixed at a 1:1 ratio with the DNA and incubated for 15 min at room temp (rt). This DNA-lipofectamine mixture was added to the adherent trophozoites in EM and incubated for 4 h at 30 °C. The EM and transfection mixture was removed by pipetting and replaced with SM with Pen-Strep and incubated for 1 day at 30 °C. To initiate selection one day after transfection, the spent media was decanted, new media (SM) with G418 (12 ug/mL G418) was added, and the cultures were incubated for another 3 days at 30 °C. The selection media was exchanged every 3 days until adherent trophozoites began to proliferate.

Mitochondria were isolated from transfected *Acanthamoeba* cell lines following the same protocol stated above. Isolated mitochondria pellets were thawed on ice and lysed in lysis buffer (50 mM Tris pH 7.5, 150mM NaCl, 0.3% DDM, 500 μM PMSF, and Roche complete protease inhibitor cocktail tablet) by gentle pipetting and left on ice for 20 min. The solubilized fraction was collected by centrifugation (20,000 x g for 10 min) and then incubated in Pierce^™^ Anti-HA Magnetic beads (TFS) for 2 hours at 4 °C. Beads were then washed twice in wash buffer (50 mM Tris pH 7.5, 150 mM NaCl, 0.03% DDM, 500 μM PMSF, and Roche complete protease inhibitor cocktail) and the sample (ATP synthase-MDH, ATP synthase-MDH_H294A_, and ATP synthase-MDH_ΔNTD_) was eluted in 500uL of wash buffer with HRV 3C Protease by incubation for 1 hour at 4 °C. This elution was then incubated with HisPur^™^ Ni-NTA Magnetic Beads (TFS) for 10 min at 4 °C to remove the protease from the sample. The flow through was collected and used for *in vitro* steady state kinetic assays.

#### Confocal microscopy to validate localization

Cells were prepared and imaged following the same protocol detailed in Stefely et al., co-submitted^43^. In short, cells were cultured in a 8-well chamber slide system (Nunc^™^ Lab-Tek^™^ II CC2^™^; Thermo Cat# 154941PK). Each well was seeded with 1×10^4^ cells in SM and incubated at 30 °C for 12–24 hours. The culture medium was then aspirated and the cells were immediately fixed with 4% formaldehyde in PBS. After 30 min the fixative was aspirated and the cells were washed with PBS for 1 min and then 1% BSA in PBS for another minute. Then the cells were permeabilized with 0.1% (w/v) Triton-X-100 in PBS for 5 min. The detergent solution was removed and replaced with blocking solution (1% BSA in PBS) and incubated for 1 h at 22 °C. The blocking solution was aspirated, replaced with the appropiate primary antibody solution (e.g., Anti-HA-AF488 (mouse, 1:400, v/v) and anti-AcMICU1 (rabbit, 1:1000, v/v), diluted in PBS with BSA (1%, w/v)) and incubated under gentle rocking protected from light for 1 hour at rt. Then the cells were washed three times with 1% BSA in PBS for 10 min. The wash solution was aspirated, replaced with secondary antibody solution (e.g., 1:1000 (v/v) dilution of goat anti-Rabbit-AF647 conjugate, diluted in PBS with BSA (1%, w/v)), and incubated under gentle rocking protected from light for 1 h at 22 °C. The cells were again washed three times with 1% BSA in PBS for 10 min. The slides were mounted with Prolong Glass Antifade Mountant with NucBlue (Thermo Cat# P36981) and a glass coverslip and then stored in the dark at room temp to cure overnight

Cells were imaged with a Nikon Eclipse Ti2, Nikon AX, DUX-ST equipped with laser lines at 405, 488, 561, and 640 nm. Z-series images (stacks of ~10 images separated by ~0.1 um) were acquired. After acquisition, Nikon 3D deconvolution was applied to the Z-series. Images were corrected for background using FIJI (Image J) software, from which TIFF image files of a single plane of the Z-series were exported.

## Supplementary Material

1

2

3

4

5

6

Supplementary Datasets

- **Data S1, related to** STAR Methods: MitoCarta Tree of Life (MitoTOL) consortium membership roster and co-submitted MitoTOL manuscripts.
- **Data S2, related to** Figure 1: ATP synthase subunit sequences.
- **Data S3, related to** STAR Methods: Reconciled consensus protein phylogenies for MPPα/β and CRC1/2.
- **Data S4, related to** STAR Methods: Reconciled consensus protein phylogenies for subunit e.

## Figures and Tables

**Figure 1.**
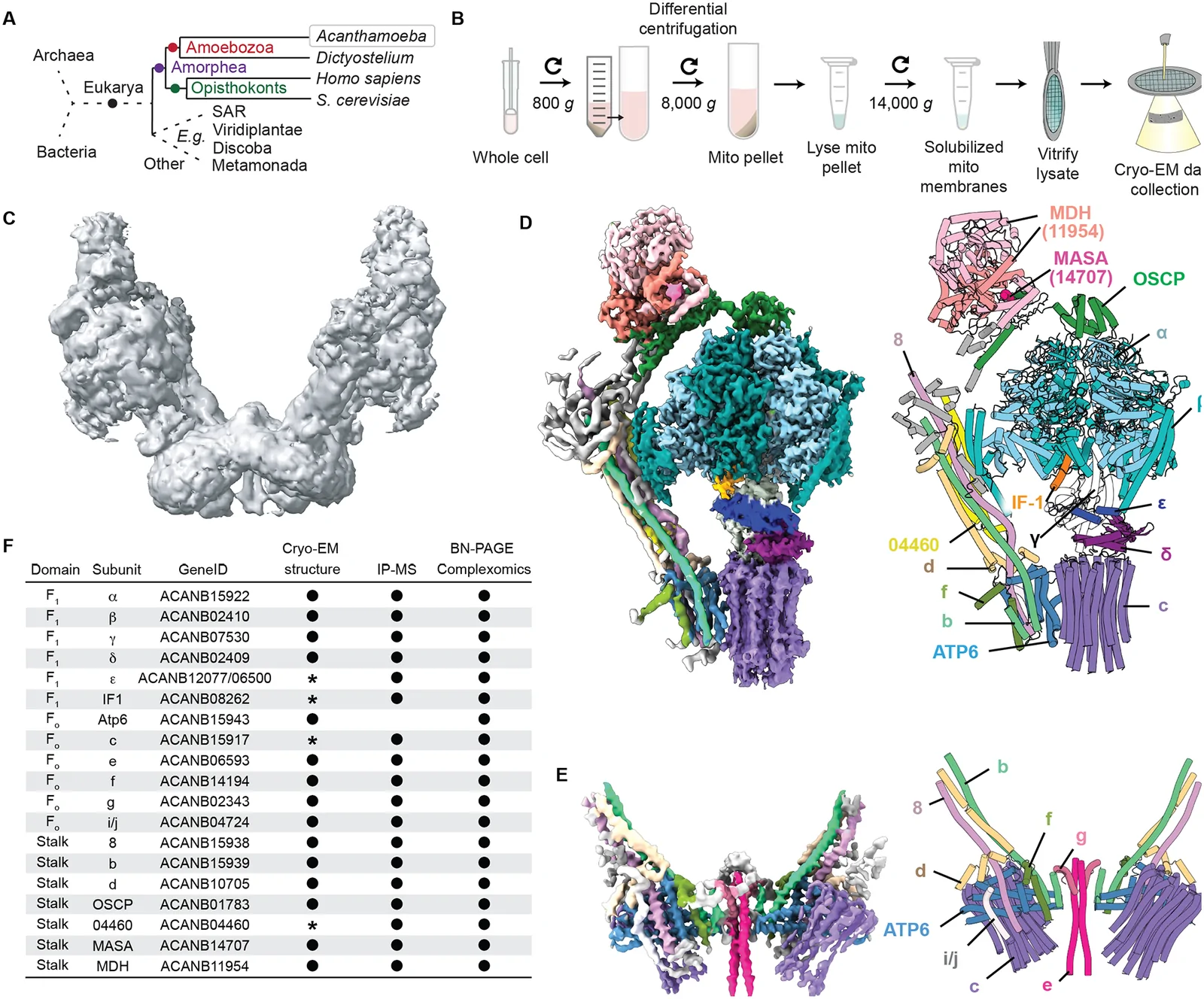
Structure of mitochondrial ATP synthase dimer from *Acanthamoeba castellanii* mitochondrial lysate. (A) Phylogenetic tree showing the relationship of *Acanthamoeba* to other eukaryotes. (B) A schematic representation of cryo-EM sample preparation. (C) The unsharpened overall map of AcATP synthase dimer. (D) Side-by-side representation of a composite map of the sharpened AcATP synthase monomer cryo-EM map colored by corresponding subunits and cartoon representation of the atomic model built into the map. (E) Side-by-side representation of a sharpened 4.2 Å map of the AcATP synthase transmembrane dimer interface colored by corresponding subunits and cartoon representation of the atomic model built into the map. (F) Subunits built into the cryo-EM map; either identified directly from the density in the cryo-EM map (*de novo,* •) or by homology (*) from a list of candidates generated by IP-MS^43^ and complexomics^43,44^.

**Figure 2.**
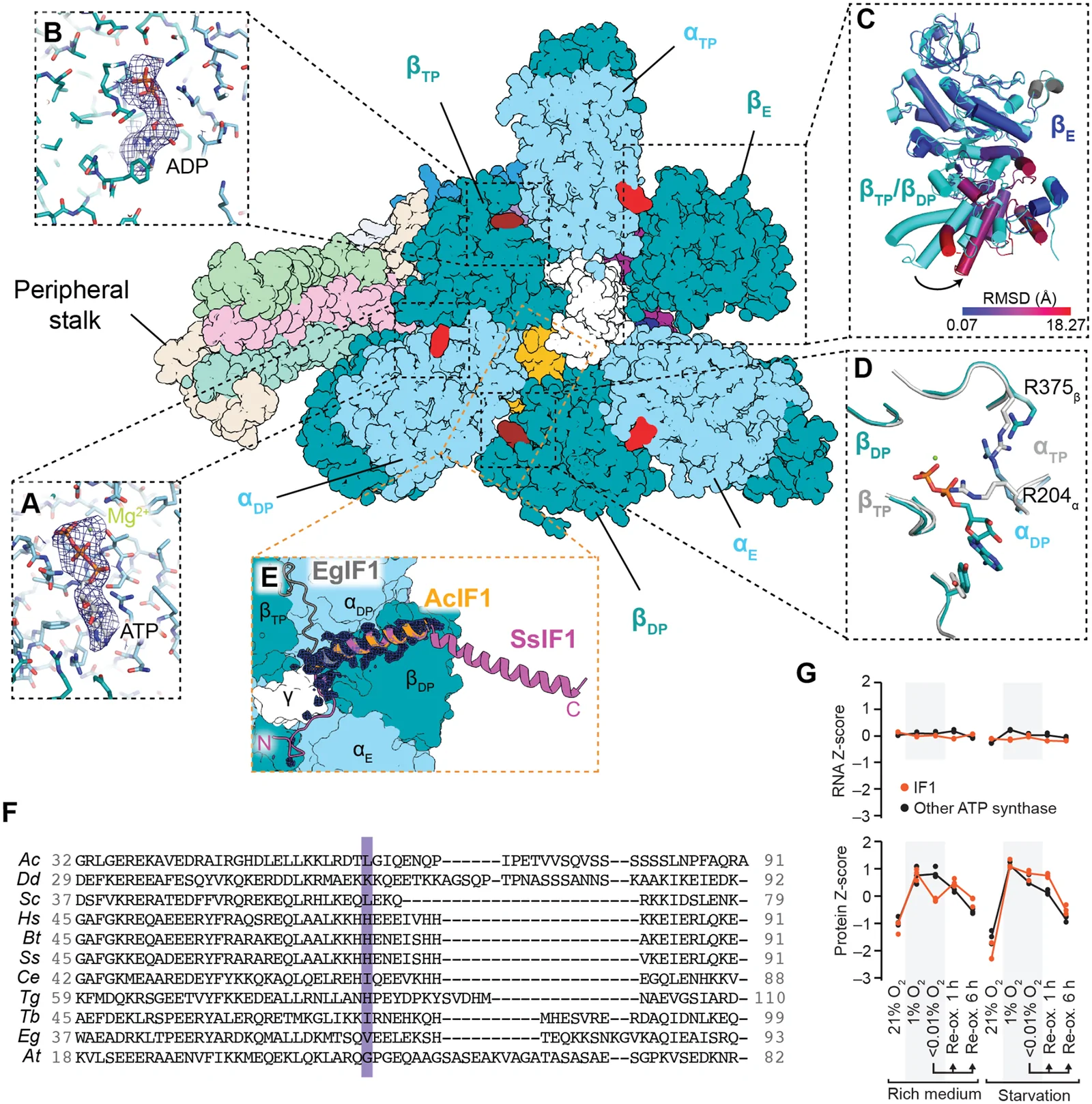
The F_1_ domain in *Ac*ATP synthase reveals an inhibited state. (A) The nucleotide binding pocket of the α subunit with the ATP molecule and magnesium ion represented as sticks. The density associated with the nucleotide is shown as a blue mesh. (B) The nucleotide binding pocket of the β subunit with an ADP molecule (sticks) built into the density (*blue*). (C) A superposition of β _TP_/β _DP_ (gray) and β _E_ (colored by RMSD (Å) from *blue* (0.07) *to red* (18.27)) revealing the conformational change in nucleotide-free β subunit. (D) A comparison of the nucleotide binding pocket in β _TP_ (*gray*) and β _DP_ (*teal*) and neighboring α subunits (α_TP_ in gray and α_DP_ in *light blue*). Key arginines from both β and α subunits (*light blue/gray*) are displayed as sticks. (E) Superposition of cartoon representation of IF1 from *Acanthamoeba* (orange), *S. scrofa* (*magenta*, PDB:6J5K), and *E. gracilis* (*gray*, PDB:6TDU) on a surface representation of the *Acanthamoeba* F_1_ domain. Density from the *Acanthamoeba* cryo-EM map is shown as a blue mesh. (F) A sequence alignment of IF1 from *Acanthamoeba*, *D. discoideum*, *S. cerevisiae*, *H. sapiens*, *B. taurus*, *S. scrofa*, *C. elegans*, *T. gondii*, *T. brucei*, *E. gracilis*, and *A. thaliana*. The pH sensing histidine in metazoans is highlighted in purple. (G) RNA and protein Z-score trajectories for IF1 (*red*) and the average of other ATP synthase subunits (*black*) across O_2_ perturbation profiling conditions (n = 3). Primary perturbation profiling data is reported in Stefely et al, co-submitted^43^.

**Figure 3.**
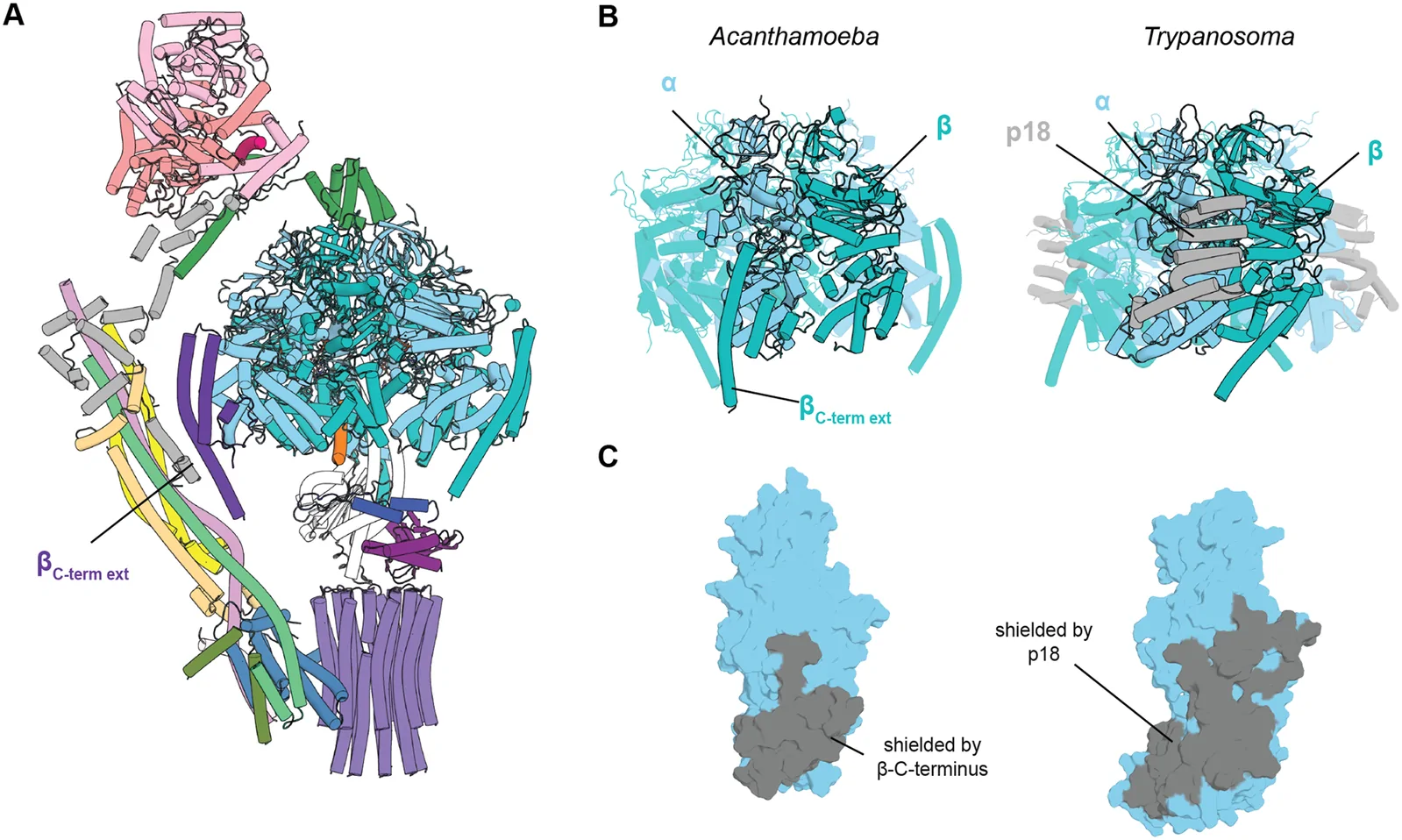
Species-specific adaptations in the F_1_ domain. (A) The ATP synthase monomer atomic model with the β subunit C-terminal extension closest to the peripheral stalk colored in *dark purple*. (B) A cartoon representation of the three copies of α (*blue*) and β subunits (*teal*) in the F_1_ region from *A. castellanii* and *T. brucei* (PDB: 8AP6). In the *T. brucei* structure an additional subunit, p18 (*gray)*, shields the α subunit. (C) Surface representation of the α subunit where the surfaces shielded by either the C-terminus of the β subunit in *A. castellanii* or p18 in *T. brucei*, are colored in *gray*.

**Figure 4.**
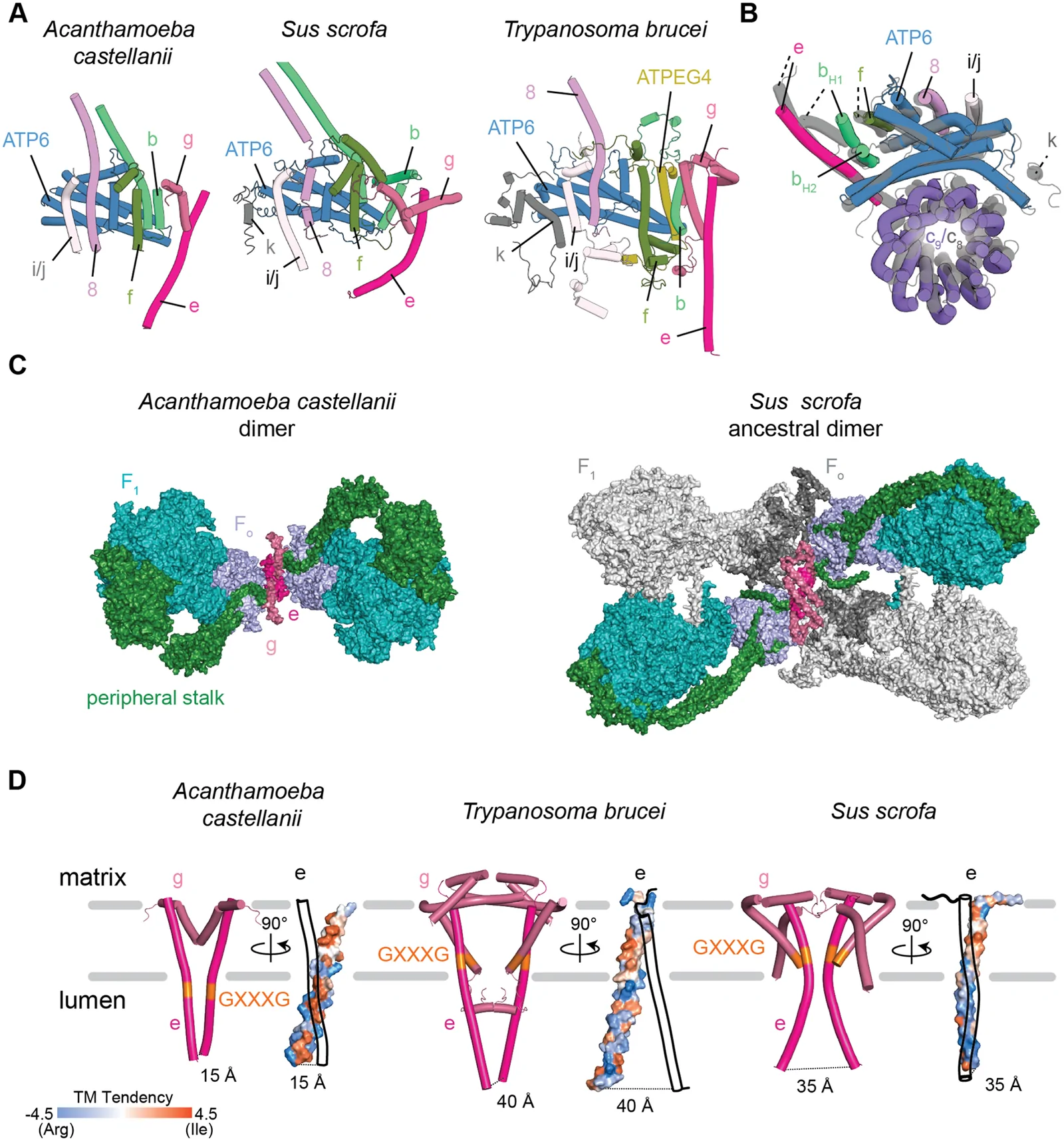
Tight packing of the F_o_ transmembrane region is reflected in the dimer interface. (A) A cartoon representation of the transmembrane segments of the subunits making up the F_o_ region for *A. castellanii*, *S. scrofa* (PDB: 6J5K), and *T. brucei* (PDB: 8AP6). Subunits are colored as in Figure 1E. An additional subunit, missing from *A. castellanii*, but present in *T. brucei* and *S. scrofa*, is colored in *gray*. (B) Top view of the F_o_ region with the c-ring. *A. castellanii* subunits are colored as in Figure 1E and labeled by solid lines and the *S. scrofa* subunits are in *gray* and labeled by dotted lines. The c-ring stoichiometry is 9 for *A. castellanii* and 8 for *S. scrofa*. (C) Surface representations of the dimeric AcATP synthase and tetrameric SsATP synthase (PDB: 6TDU). The ancestral dimerization pair are colored as such: the peripheral stalks (*green*), F_o_ (*blue*), and F_1_ (*teal*). For the mammalian tetramer, the other monomers are colored in *gray*, with the F_o_ in *dark gray* and the F_1_ in *light gray*. (D) Cartoon representation of subunits e (*magenta*) and g (*pink*) from *A. castellanii*, *T. brucei* (PDB:8AP6), and *S. scrofa* (PDB: 6TDU) ancestral dimer interface. GXXXG motifs are colored in *orange*. A 90° turn of the e subunits with one subunit surface colored by hydrophobicity (*orange* (hydrophobic) to *blue* (hydrophilic)) and the other one drawn in a black outline. Distance between the C-terminus of each pair of e subunits is indicated by a dotted line and value. The inner mitochondrial bilayer is drawn in gray lines with the matrix on top and lumen on bottom.

**Figure 5.**
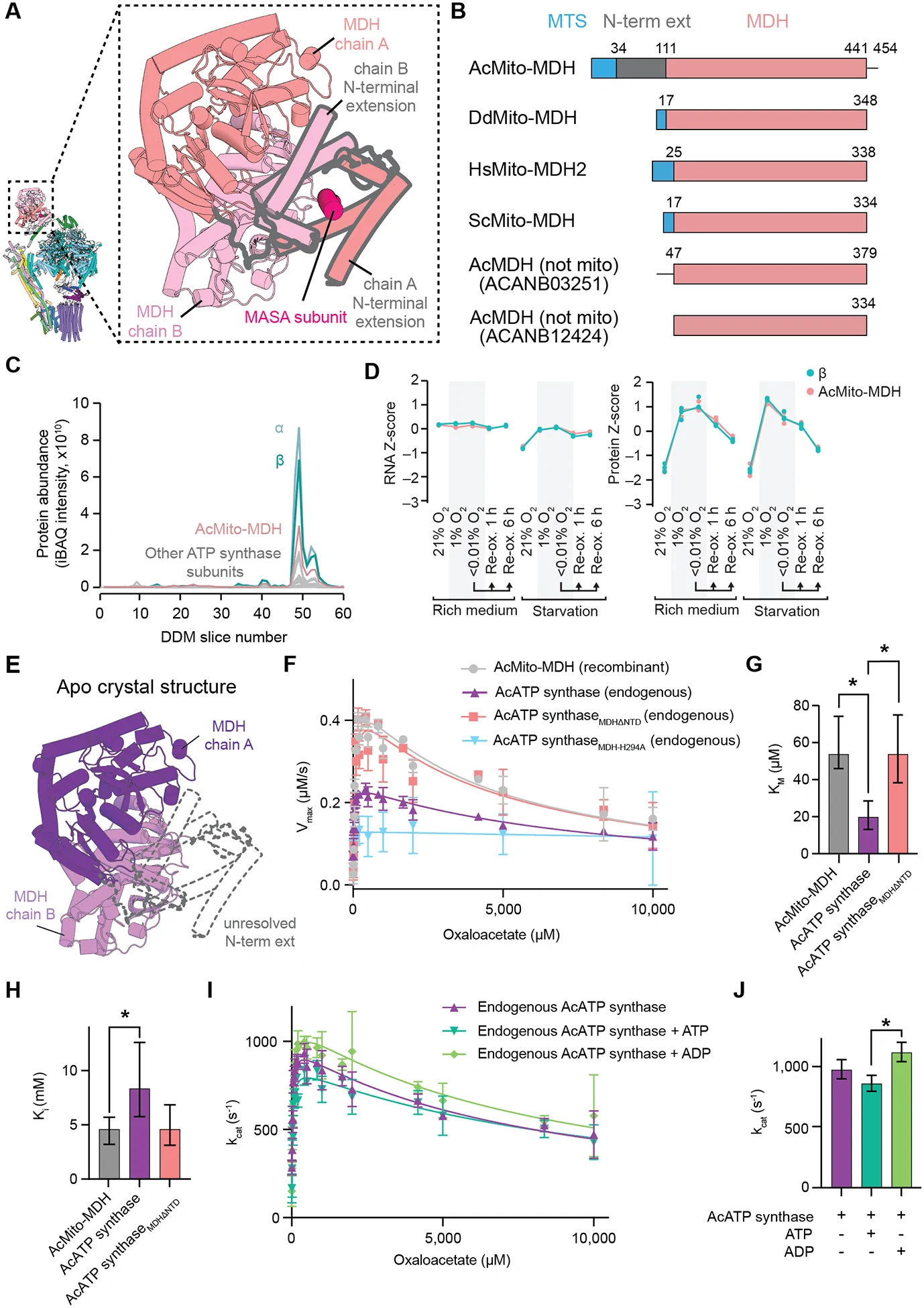
Structural integration of a mitochondrial-MDH into ATP synthase. (A) Overall structure of AcATP synthase monomer. The AcMito-MDH dimer is highlighted by a box and zoomed in image of the AcMito-MDH dimer and the MASA subunit (*hot pink)* interaction. One AcMito-MDH monomer is colored in salmon and another in pink with the three helices formed by the N-terminal extensions are outlined in gray. (B) Cartoon schematic of the sequences of MDHs from *A. castellanii*, *D. discoideum*, *H. sapiens*, and *S. cerevisiae*. Mitochondrial targeting sequences are denoted in blue, the *Acanthamoeba* N-terminal extension (N-term ext) in gray, and the MDH domain in salmon. (C) Protein abundances of *Ac*ATP synthase members in each of 60 slices of a blue native gel (BN-PAGE) with *Acanthamoeba* mitochondria solubilized in DDM. Protein abundance is based on iBAQ (intensity-based absolute quantification) analysis of MS data. Primary BN-PAGE-MS data reported in Stefely et al, co-submitted^43^. (D) Protein and RNA Z-score trajectories for mito-MDH and the ATP synthase β subunit across O_2_ and nutrient perturbation profiling conditions (n = 3 biological replicates indicated by points, lines connect the medians). Z-scores were calculated across all ten conditions shown. Original data and perturbation experiment reported in Stefely et al, co-submitted^43^. (E) A 2.3 Å crystal structure of apo AcMito-MDH with each chain colored differently (chain A: *light purple*, chain B: *dark purple*). The N-terminal extension from the cryo-EM structure is overlayed as a dotted outline. (F) Enzyme velocity of recombinant AcMito-MDH alone (*grey*) and endogenous AcATP synthase complexes *(magenta*) and mutants, the catalytically dead H294A AcMito-MDH mutant (AcATP synthase_MDH-H294A_, *light blue*) and the MDH-ΔNTD mutant (AcATP synthase_MDHΔNTD_, *pink*), are shown as oxaloacetate concentration was varied. The rate of oxidation of NADH to NAD^+^ was measured by monitoring change in absorbance at 340 nm during the reaction and converted to μM/sec. (G) Bar plot of calculated K_M_ values for recombinant AcMito-MDH (*grey*), endogenous AcATP synthase (*magenta*), and endogenous AcATP synthase_MDHΔNTD_ (*pink*). Significance based on no overlap of the 95% CI is denoted by *; n = 6. (H) Bar plot of calculated K_i_ values for recombinant AcMito-MDH (*grey*), endogenous AcATP synthase (*magenta*), and AcATP synthase_MDHΔNTD_ (*pink*). Significance based on no overlap of the 95% CI is denoted by *; n = 6. (I) Enzyme velocity of endogenous AcATP synthase alone (*magenta*) and with ATP (*teal*) or ADP (*green*), are shown as oxaloacetate concentration was varied. The rate of oxidation of NADH to NAD^+^ was measured by monitoring change in absorbance at 340 nm during the reaction and converted to μM/sec. (J) Bar plot of apparent k_cat_ values for endogenous AcATP synthase alone (*magenta*), and with ATP (*teal*) or ADP (*green*). Significance based on no overlap of the 95% CI is denoted by *; n = 6.

**Figure 6.**
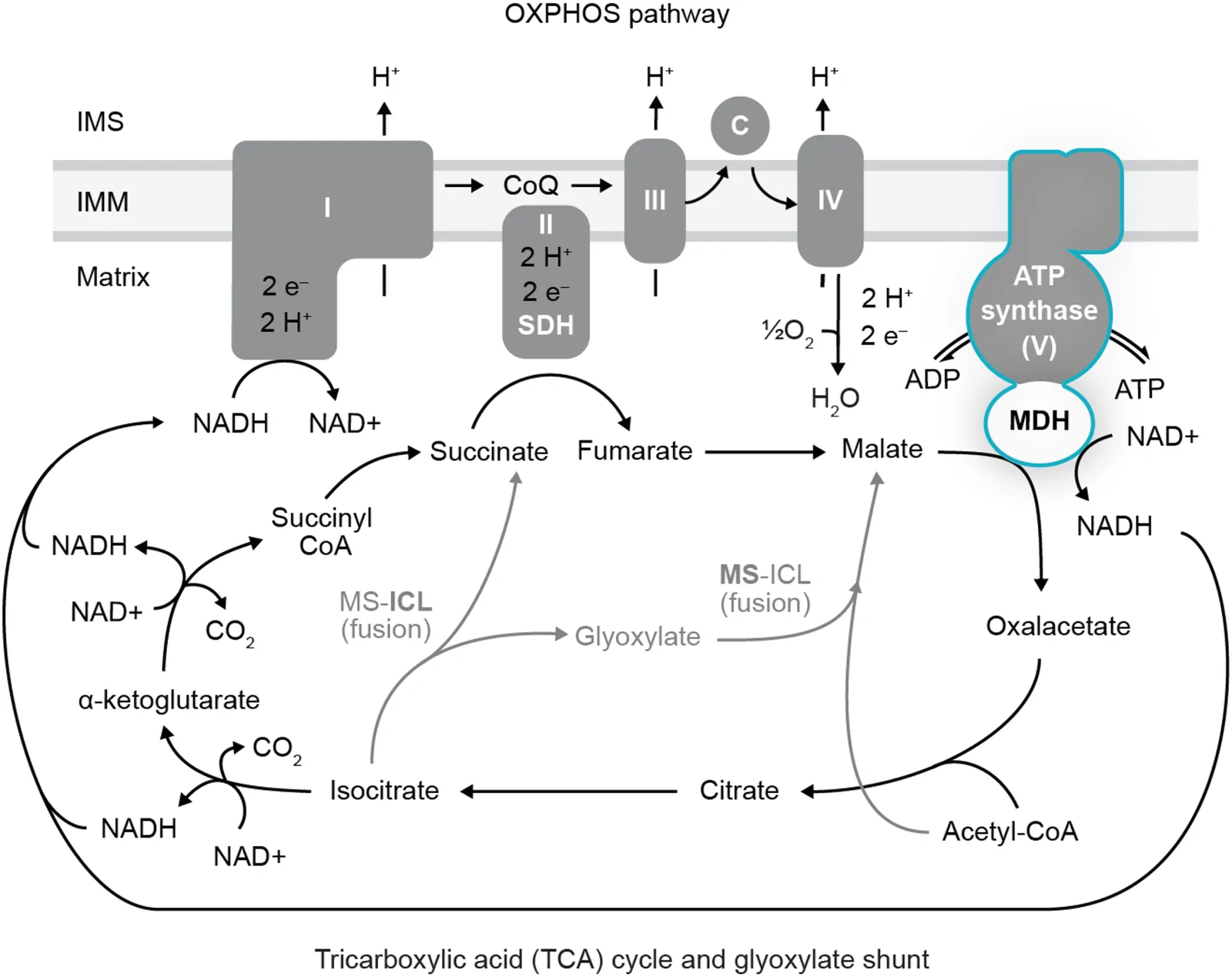
A model for structurally linked metabolic and respiratory pathways in *Acanthamoeba* An overall schematic of novel features in OXPHOS, TCA cycle, and glyoxylate shunt in *Acanthamoeba*. The TCA cycle enzyme, MDH is integrated into the OXPHOS component, ATP synthase or CV, resulting in a species-specific linkage between the two bioenergetic pathways. Additionally, there is evidence of a glyoxylate shunt (*gray*) in the mitochondria of *Acanthamoeba*^43,59^. In the glyoxylate shunt, many of the TCA enzymes are bypassed with the conversion of isocitrate to glyoxylate and succinate by isocitrate lyase (ICL) and subsequently malate synthase (MS) catalyzes the conversion of glyoxylate and Acetyl-CoA into malate and CoA. In *Acanthamoeba*, these two enzymes ICL and MS are fused resulting in a single enzyme, MS-ICL^43,59^.

**Table 1:** Cryo-EM statistics.

	ATP synthase dimer	ATP synthase monomer	ATP synthase F_1_	ATP synthase MDH-OSCP	ATP synthase F_o_ dimer	Complex III	mito-Hsp60
**Data collection & processing**							
Microscope	FEI Titan Krios					FEI Titan Krios	FEI Titan Krios
Voltage (kV)	300					300	300
Camera	K3 Gatan Direct Detector					K3 Gatan Direct Detector	K3 Gatan Direct Detector
Energy filter	BioQuantum Gatan Image Filter					BioQuantum Gatan Image Filter	BioQuantum Gatan Image Filter
Energy filter slit width (eV)	20					20	20
Magnification (nominal)	105,000					105,000	105,000
Defocus range (*μ*m)	−0.6 to −2.0					−0.6 to −2.0	−0.6 to −2.0
Calibrated pixel size (Å/pix)	0.41					0.41	0.41
Electron exposure (e^−^/Å^2^)	46.44 to 54.1					46.44 to 54.1	46.44 to 54.1
Exposure rate (e^−^/Å^2^/frame)	0.92 to 1.06					0.92 to 1.06	0.92 to 1.06
Number of frames per movie	40 to 50					40 to 50	40 to 50
Automation software	EPU					EPU	EPU
Number of micrographs	68,290					68,290	68,290
Final particle images (no.)	11,338	10,863	10,863	10,863	11,768	5,208	1,154
Map resolution (Å, FSC=0.143)		4.2	3.8	4.3	4.1	3.86	3.9
Symmetry	C1	C1	C1	C1	C2	C2	D7
cFar		0.57	0.59	0.36	0.49	0.61	0.68
**Model building and fitting**							
Software, building (*Model Angelo*)			1.0.14	1.0.14	1.0.14	1.0.14	1.0.14
Software, fitting (*ChimeraX*)	ChimeraX-1.9	ChimeraX-1.9	ChimeraX-1.9	ChimeraX-1.9	ChimeraX-1.9	ChimeraX-1.9	ChimeraX-1.9
**Refinement**							
Software (*phenix.real_space_refine*)		PHENIX 1.21.2–5419	PHENIX 1.21.2–5419	PHENIX 1.21.2–5419	PHENIX 1.21.2–5419	PHENIX 1.21.2–5419	PHENIX 1.21.2–5419
Resolution of unmasked reconstructions (Å, FSC=0.143)		4.1	3.6	4.2	4.3	4.1	3.9
Resolution of masked reconstructions (Å, FSC=0.143)		4.0	3.6	4.1	4.2	4.4	4.0
Correlation coefficient (CC_*mask*_)		0.60	0.8	0.75	0.73	0.77	0.82
Map sharpening *B* factor (Å^2^)							
Model composition							
Non-hydrogen atoms		48,010	25,855	7,698	10,124	26,321	46,774
Protein residues		6,185	3,328	1,001	1,218	3272	6,342
Ligand		8	8	0	0	4	0
*B* factors (Å^2^)		min/max/mean	min/max/mean	min/max/mean	min/max/mean	min/max/mean	min/max/mean
Protein		0.0/550.09/109.49	26.89/155.26/97.05	30.0/225.01/515.55	83.15/295.69/156.46	30.00/154.30/111.90	55.77/177.85/95.98
Ligand		79.55/109.01/98.17	75.70/103.80/99.64			0.0/10.75/5.43	
Bond RMSD							
Bond lengths (Å) (# 4*σ)*		0.005 (0)	0.004 (0)	0.004 (0)	0.003 (0)	0.005 (0)	0.005 (1)
Bond angles (°) (# 4*σ)*		0.953 (0)	0.946 (1)	0.888 (0)	0.530 (1)	0.617 (1)	0.566 (0)
**Validation**							
MolProbity score		1.89	1.33	1.38	1.66	1.59	1.46
Clashscore		8.64	3.92	4.85	8.75	8.06	5.19
rotamer outliers (%)		0.02	0.29	0	0	0.22	0.02
C*β* outliers (%)		0	0	0	0	0	0
CaBLAM outliers (%)		1.11	0.95	0.83	0	1.37	1.27
EM Ringer			1.25	0.54	0.17	1.90	2.17
Ramachandran plot							
Favored (%)		95.28	97.18	97.35	96.86	97.19	96.88
Allowed (%)		4.69	2.79	2.65	3.14	2.81	3.12
Disallowed (%)		0.03	0.03	0	0	0	0

**Table:** KEY RESOURCES TABLE

REAGENT OR RESOURCE	SOURCE	IDENTIFIER
**Antibodies**		
Monoclonal Anti-FLAG M2 antibody (mouse)	Sigma	F3165-.2MG
Mouse IgG HRP Linked Whole Ab	Sigma / Cytiva	GENA931-1ML
**Bacterial strains**		
One Shot ^®^ Top10 Chemically Competent *E. coli*	Invitrogen	C404003
NiCo21(DE3) Competent *E. coli*	NEB	C2529H
**Chemicals, peptides, and recombinant proteins**		
AcMito-MDH (ACANB11954), recombinant protein purified from *E. coli*	This study	
AcMito-MDH-H291A, recombinant protein from *E. coli*	This study	
AcMito-MDH with ACANB14707, recombinant protein complex from *E. coli*	This study	
6xHis-ULP1, recombinant protein, purified from *E. coli*	This study	
Benzonase (Purity >99%)	Millipore	70664-3
β-Nicotinamide adenine dinucleotide, reduced disodium salt hydrate	Sigma	N8129
n-Dodecyl β-D-maltoside	Sigma	D5172
Phenylmethylsulfonyl fluoride (PMSF)	Sigma / Roche	10837091001
oxalacetic acid, 90+%	Thermo	AA1578909
Terrific Broth, granulate	Research Products International	T15100-2000.0
Beta-meraptoethanol	Sigma	M6250
Ni-NTA Agarose	Qiagen	30230
Isopropyl β-D-1-thiogalactopyranoside (IPTG) >=99%	Sigma	I6758
Dithiothreitol, >99.5%, Molecular Biology Grade UltraPure	Thermo	J15397.06
**Deposited data**		
AcATP synthase monomer composite cryo-EM map	This study	EMD-74152
AcATP synthase monomer cryo-EM map	This study	EMD-74153
AcATP synthase transmembrane dimer interface cryo-EM map	This study	EMD-74154
AcATP synthase F_1_ domain cryo-EM map	This study	EMD-74156
AcATP synthase MDH-OSCP region cryo-EM map	This study	EMD-74155
*Acanthamoeba* Complex III cryo-EM map	This study	EMD-74150
*Acanthamoeba* mitochondrial Hsp60 cryo-EM map	This study	EMD-74149
AcATP synthase monomer composite atomic model	This study	PDB:9ZFT
AcATP synthase transmembrane dimer interface atomic model	This study	PDB:9ZFU
AcATP synthase F_1_ domain atomic model	This study	PDB:9ZFV
AcATP synthase MDH-OSCP atomic model	This study	PDB:9ZFW
Atomic model of *Acanthamoeba* complex III intermediate	This study	PDB:9ZFS
Atomic model of *Acanthamoeba* mitochondrial Hsp60	This study	PDB:9ZFR
AcMito-MDH crystal structure	This study	PDB:9ZFX
**Experimental models: Organisms/strains**		
*Acanthamoeba castellanii* strain Neff, wild type	ATCC	30010
**Recombinant DNA**		
6xHis-SUMO-*Ac*Mito-MDH (ACANB11954)	This study	N/A
6xHis-SUMO-*Ac*Mito-MDH (ACAN11954) H291A mutant	This study	N/A
3xFLAG-GGGS-ACANB14707	This study	N/A
pET33b-6xHis-ULP1	Bil Clemons, Caltech	N/A
Plasmid: FDp184_AcMito-MDH-[C3]-His6-HA (ACANB11954) [AcNdufs4 promoter]	This study	N/A
Plasmid: TMIp191_AcMito-MDH-[C3]-His6-HA (ACANB11954) [own genomic promoter]	This study	N/A
Plasmid: TMIp192_AcMito-MDH[H294A]-[C3]-His6-HA (ACANB11954) [own genomic promoter]	This study	N/A
Plasmid: TMIp194_AcMito-MDH[ΔNTD]-[C3]-His6-HA (ACANB11954) [own genomic promoter]	This study	N/A
**Software and algorithms**		
Excel for Mac	Microsoft	Version 16.102.3
FIJI (Image J)	National Institutes of Health	
ChimeraX	UCSF/National Institutes of Health	1.9
PyMOL	Schrödinger	3.1.5.1
EPU	Thermo	2.12.1.2782REL
XDS	MPI for Medical Research Heidelberg	20241002_x86
Prism	GraphPad	Version 10
autoProc	Global Phasing	v1.0.5
SBGrid	SBGrid consortium	
MolProbity	Duke University	
cryoSPARC	Structura Biotechnology	v4.7.0
Phenix	The Phenix Industrial Consortium/National Insititues of Health	v1.21.2
COOT	University of Cambridge	0.9.8.95
ModelAngelo	MRC Laboratory | Scheres Lab	1.0
AlphaFold3	DeepMind	
**Other**		
GenCatch ^™^ Plasmid DNA Mini-Prep Kit	Epoch life sciences	2160250
Q5 Site-Directed Mutagenesis Kit	New England Biolabs	E0554S
Superose 6 10/300	GE Healthcare Inc	29091596
Quantifoil ^®^ Cu-300 mesh R1.2/1.3	Electron Microscopy Sciences/Quantifoil	Q350CR1.3
Pelco easiGlow^™^ Emeritech K100X	Ted Pella	
FEI Vitrobot Mark v4	FEI/Thermo	
FEI Titan Krios	FEI/Thermo	
K3 Camera	Gatan	
BioTek Cytation5 imaging reader	Aligent	07-200-98
Corning^™^ Clear Polystyrene 96-Well Microplates	Fisher Scientific	

## Highlights

- Multiple cryo-EM structures from mitochondrial lysate
- Structural analysis of *Acanthamoeba* ATP synthase, Hsp60, and complex III
- Structural evidence for a physical link between the TCA cycle and OXPHOS
- Biochemical characterization of mitochondrial MDH in complex with ATP synthase

Acanthamoeba castellanii, a pathogenic amoeba belongs to an evolutionary branch distinctly removed from humans and fungi. Cryo-EM of the organism’s ATP synthase enzyme using raw mitochondrial lysates reveals an unexpected metabolic adaptation that allows it to wire the TCA cycle directly into the ATP-making apparatus itself.
