# Supplementary material for: Acanthamoeba ATP synthase structure reveals the TCA cycle is tethered to OXPHOS
Source: Cell. Author manuscript; Available in PMC 2026 Oct 2. (PMC13632647; doi:10.1016/j.cell.2026.08.057)
Supplement: Supplementary file 1 [file NIHMS2208837-supplement-6.pdf]

SUPPLEMENTAL FIGURES

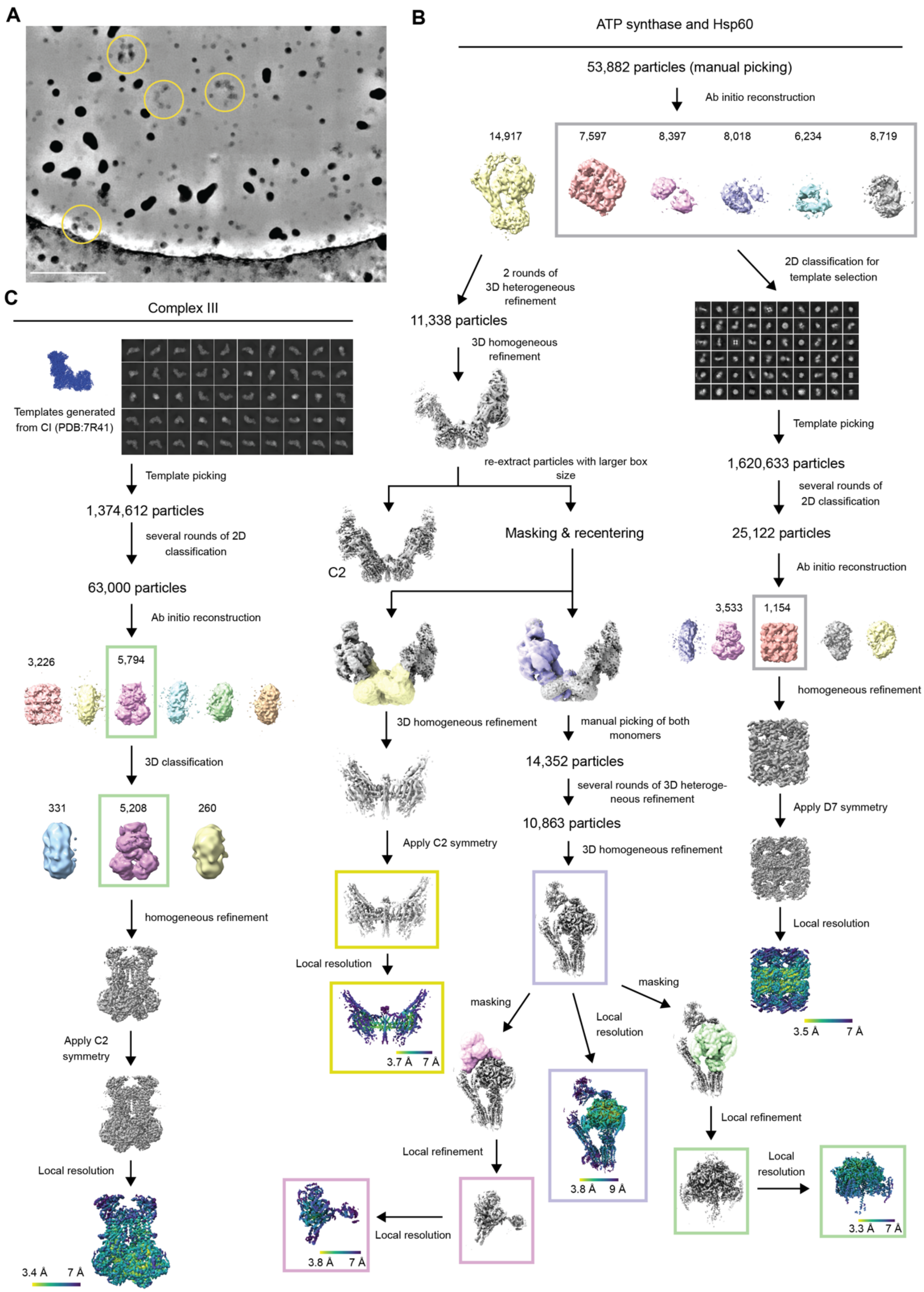

A
B
ATP synthase and Hsp60
53,882 particles (manual picking)
Ab initio reconstruction
14,917
7,597
8,397
8,018
6,234
8,719
2 rounds of 3D heterogeneous refinement
2D classification for template selection
11,338 particles
3D homogeneous refinement
Template picking
re-extract particles with larger box size
1,620,633 particles
several rounds of 2D classification
C2
Masking & recentering
25,122 particles
Ab initio reconstruction
3,533
1,154
homogeneous refinement
3D homogeneous refinement
manual picking of both monomers
14,352 particles
several rounds of 3D heteroge-neous refinement
Apply D7 symmetry
10,863 particles
Apply C2 symmetry
3D homogeneous refinement
Local resolution
Local resolution
masking
masking
Local resolution
3.5 Å
7 Å
3.7 Å
7 Å
Local refinement
Local refinement
Local resolution
3.8 Å
9 Å
3.8 Å
7 Å
Local resolution
3.3 Å
7 Å
C
Complex III
Templates generated from CI (PDB:7R41)
Template picking
1,374,612 particles
several rounds of 2D classification
63,000 particles
Ab initio reconstruction
3,226
5,794
3D classification
331
5,208
260
homogeneous refinement
Apply C2 symmetry
Local resolution
3.4 Å
7 Å

Figure S1. Processing workflow of cryo-EM data to resolve maps for ATP Synthase, mito-Hsp60 and complex III, related to Figure 1.

(A) A representative aligned micrograph from the data collection with ATP synthase particles highlighted with yellow circles. Scale bar represents 100 nm.

(B) Processing of data to obtain ATP synthase and mito-Hsp60 cryo-EM maps through cryoSPARCv4.6.1.[1] Masks are overlayed on maps used to generate focused maps (grey) in colors (*yellow, purple, pink,* and *green*) for ATP synthase. Local resolution is projected onto maps of the ATP synthase monomer (*purple*), ATP synthase F_1 domain (*green*), ATP synthase MDH-OSCP domain (*pink*), ATP synthase F_o dimerization domain (*yellow*), and mito-Hsp60. Resolution is plotted on the viridis color map from highest to lowest resolution (*yellow* to *purple*).

(C) Processing of data through cryoSPARCv4.6.1[1] for complex III. Local resolution is projected onto maps for complex III. Resolution is plotted on the viridis color map from highest to lowest resolution (*yellow* to *purple*).

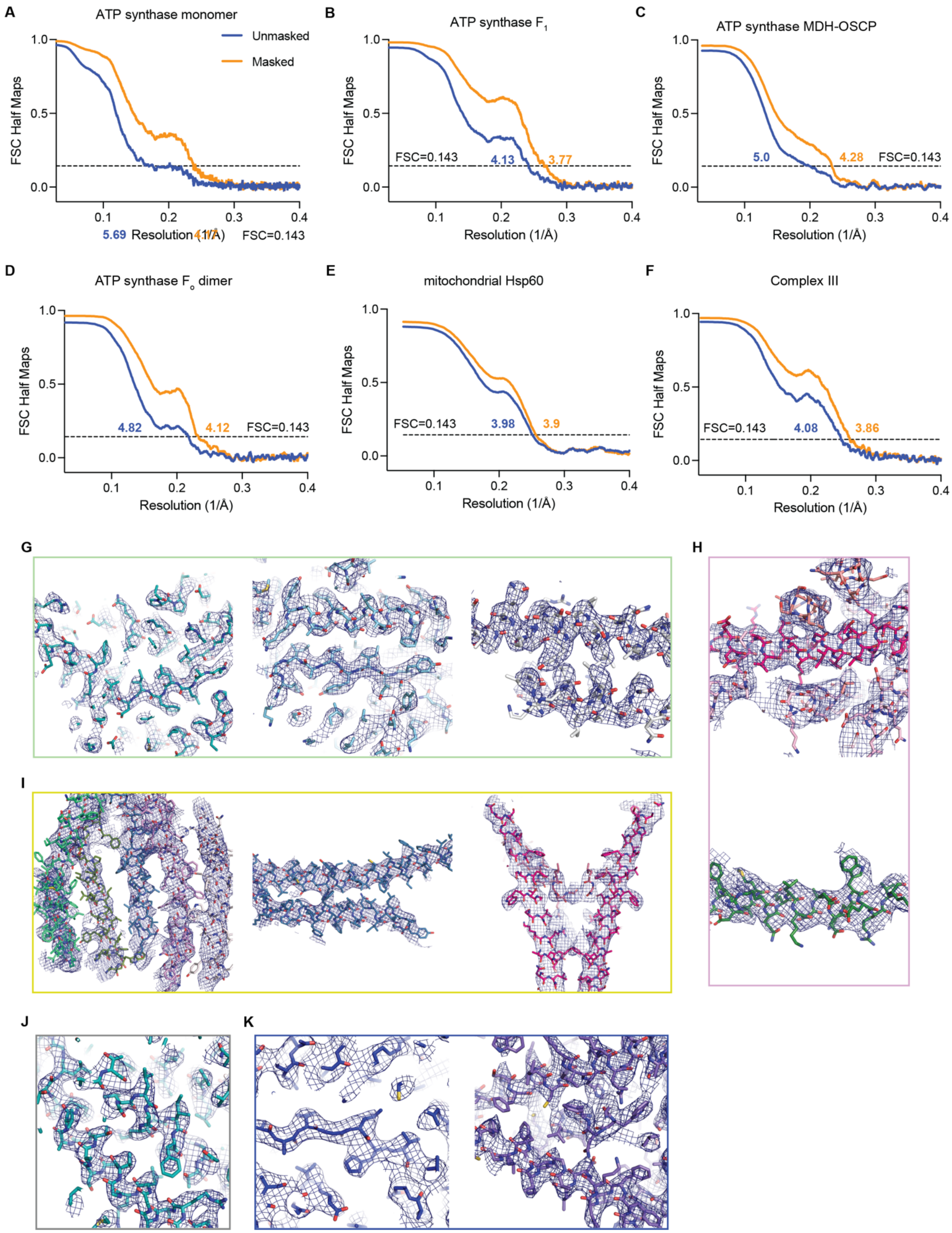

Figure S2. SPA map and model quality, related to Figure 1.

(A-F) The Gold-standard Fourier Shell Correlation curves for 'masked' (*orange*) and 'unmasked' (*blue*) half maps used

to build the (A) ATP synthase monomer, (B) ATP synthase F_1, (C) ATP synthase MDH-OSCP, (D) ATP synthase F_o dimer, (E) mitochondrial Hsp60, and (F) complex III maps from phenix v1.21.2[2]. Resolution was determined using an FSC = 0.143 criterion.

(G-K) Representative density of (G) ATP synthase F_1 (from left to right: α, β, and γ subunits), (H) ATP synthase MDH-OSCP (MDH-MASA interaction (top) and OSCP (bottom)), (I) ATP synthase F_o dimer (from left to right: TMS segments, ATP6, and subunits e and g), (J) Hsp60, and (K) complex III (UQCRC2 (right) and MT-CYB (left)). ATP synthase subunits are colored as they are in Figure 1.

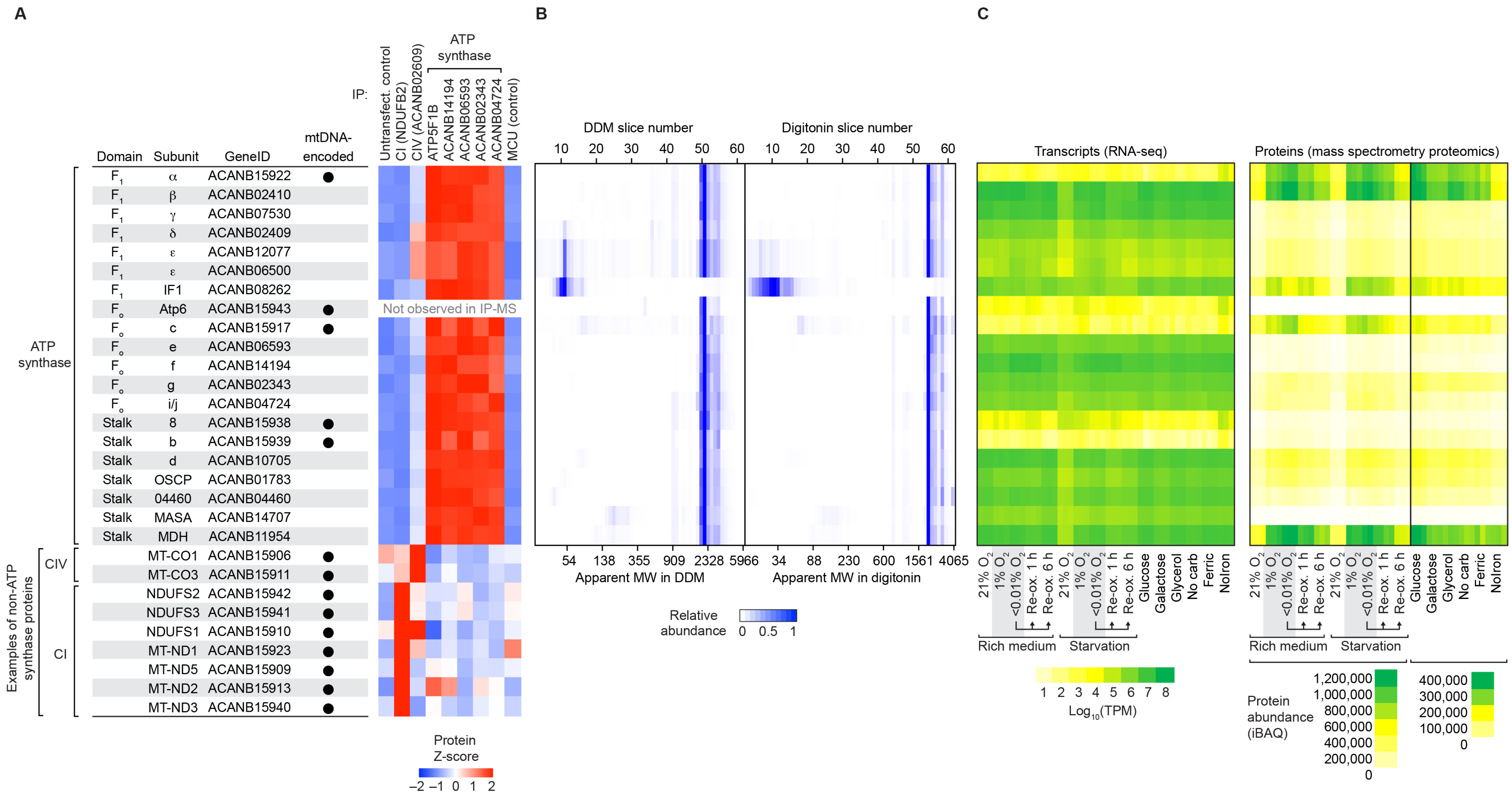

Figure S3. MS techniques to identify and track the perturbational profile of ATP synthase subunits, related to Figure 1 & 5.

(A) Summary table and heatmap of relative protein abundances (Z-scores) in immunoprecipitation-mass spectrometry (IP-MS) of complex I (CI), complex IV (CIV), and ATP synthase, highlighting all AcATP synthase subunits and a select set of mtDNA-encoded proteins in CI and CIV as controls. Primary IP-MS data reported in Stefely et al., co-submitted[3].

(B) Relative protein abundances (based on mass spectrometry (MS) proteomics) of AcATP synthase members in each of 60 slices of a blue native gel (BN-PAGE) with *Acanthamoeba* mitochondria solubilized in 0.3% DDM or 1% digitonin. Primary BN-PAGE-MS data reported in Stefely et al., co-submitted.[3]

(C) Summary of RNA-seq transcriptomics and mass spectrometry (MS) proteomics profiling data for *Ac*ATP synthase subunits across a set of perturbations of O_2 and nutrients (primary data reported in Stefely et al., co-submitted[3]). TPM, transcripts per million; mtDNA, mitochondrial DNA; Re-ox., re-oxygenation for the indicated period (1 h or 6 h) after a 24-hour period of near-anoxia (<0.01% O_2). The RNA-seq samples were analyzed together as a batch, so TPMs are directly comparable across this entire set. The MS proteomics for the O_2 perturbations was run separately from the MS proteomics for the perturbations of carbohydrate

and iron source, so separate iBAQ scales are shown for each of these sets.

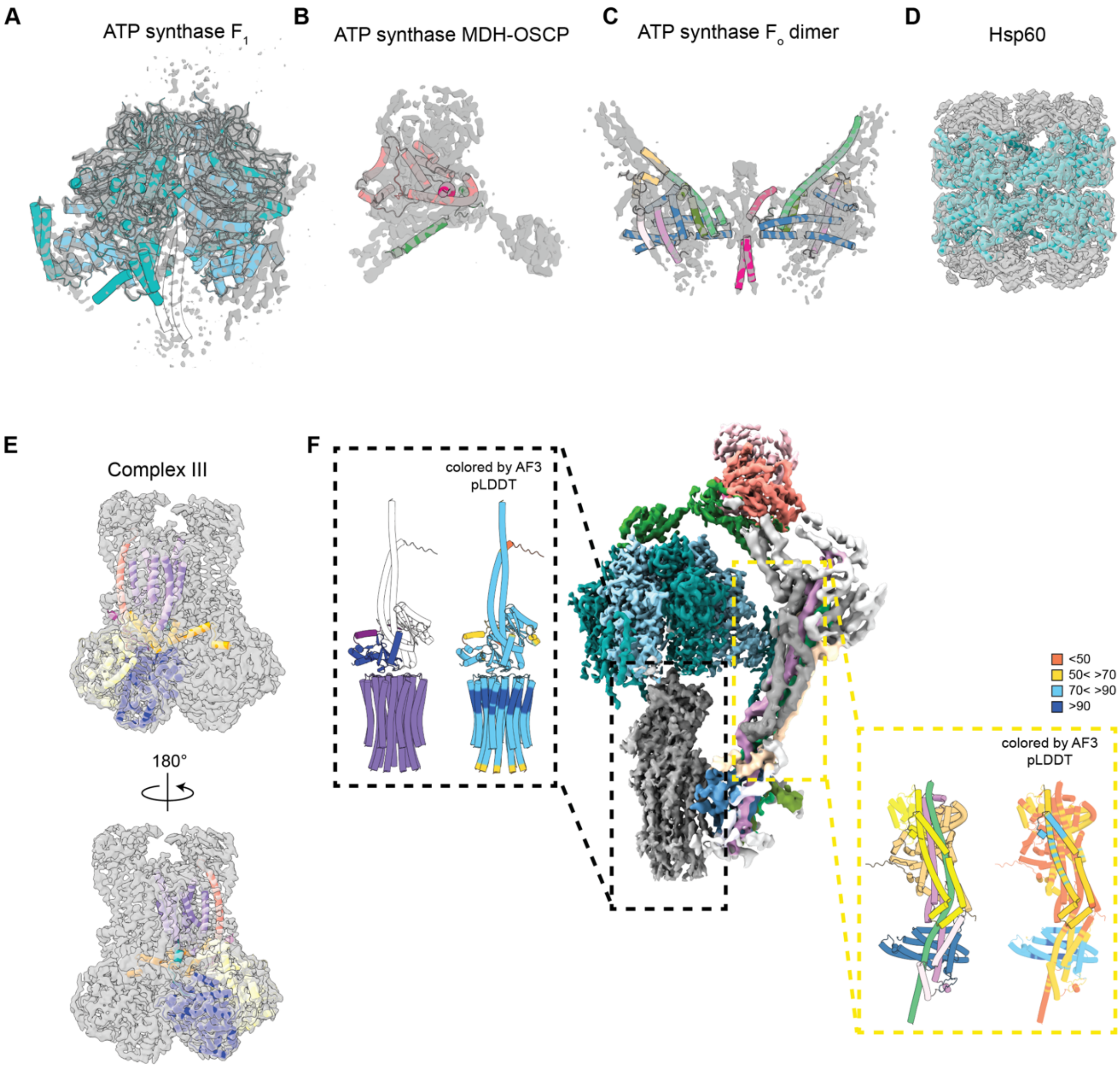

Figure S4. Automated and manual model buildings, related to Figure 1.

(A-E) Cryo-EM density maps (*grey)* with peptides built by ModelAngelo[4] resulted in hits in the *Acanthamoeba* proteome for (C) ATP synthase F_1, (D) ATP synthase MDH-OSCP, (E) ATP synthase F_o dimerization, (D) mitochondrial Hsp60, and (E) complex III maps. Peptides are colored based on the corresponding subunits as colored in **Figure 1D,E**.

(F) A composite map of the ATP synthase monomer with regions identified *de novo* by ModelAngelo[4] colored as in **Figure 1D**. Subunits not identified *de novo* are colored in *grey*. The corresponding AlphaFold3 predictions[5] are boxed for subunits c, δ, and ε and ACANB04660 (*yellow*). All subunits are colored as they are in **Figure 1D**. Models are also colored by Alphafold3[5] plDDT values.

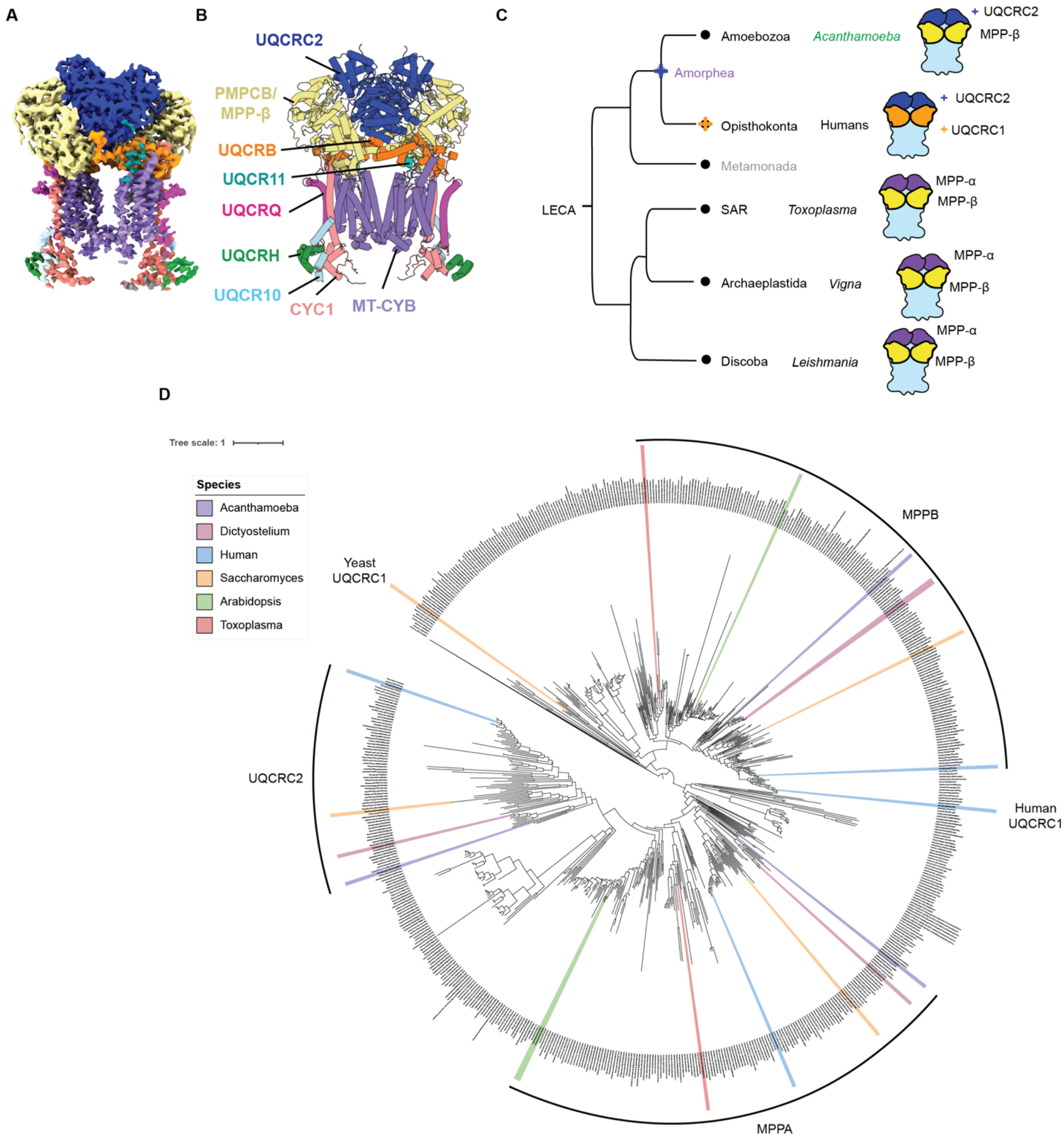

Figure S5. Cryo-EM structure of complex III reveals a unique subunit composition of the matrix facing region, related to Figure 1.

(A) A sharpened 3.8 Å map of *Acanthamoeba* complex III colored by conserved subunits

(B) A cartoon representation of the atomic model of $AcCIII_2$.

(C) Phylogenetic tree with solved structures of complex III from various eukaryotic groups, highlighting UQCRC1/MPP-β (*orange* & *yellow*, respectively) and UQCRC2/MPP-α (*blue/green* & *purple*, respectively) homologs revealing one duplication event in Amorphea of UQCRC2 (*blue* plus sign) and one duplication even in Opisthokonta of UQCRC1 (*orange* plus sign). This explains why *Acanthamoeba* $CIII_2$ possess two copies of UQCRC2 and MPP-β, a combination not previously observed.

(D) Phylogeny of MPP-α/β and UQCRC1/2 reveals a single duplication event of UQCRC2 from MPP-α.

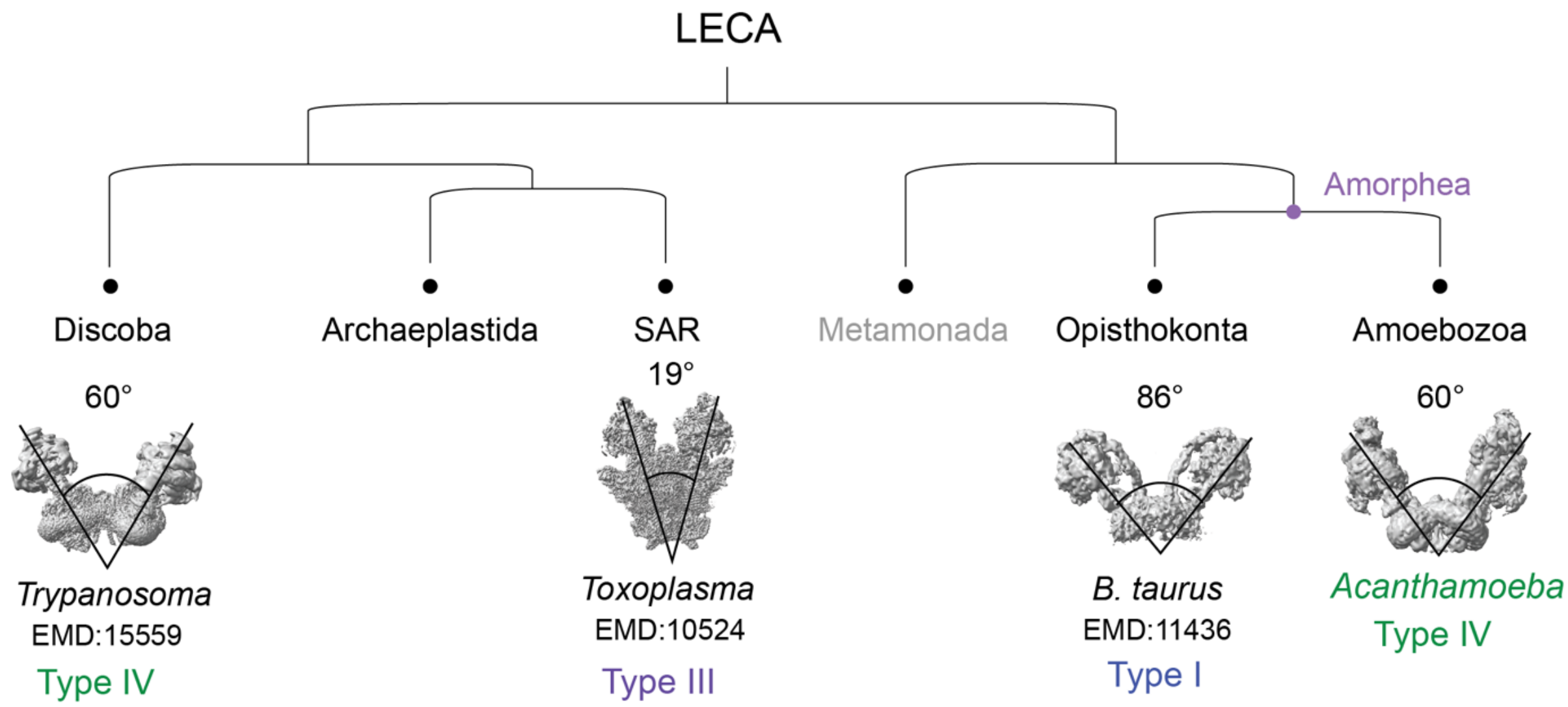

Figure S6. AcATP synthase adopts a Type IV oligomerization architecture, related to Figure 1 & 4.
A phylogenetic tree of representative solved structures of ATP synthase dimers from different eukaryotic supergroups. Dimer angles and oligomeric assembly types are annotated.

```
 A. castellanii  1 MMR--------RAATRSALCR-----PSFAPTAAR------------------------GYSSVLEAGGRLGEREKAVEDRAIRGHDLELLKKLRDTLG 62
 D. discoideum   1 MS----------SSVSKLLKLTRA---SMIAAPKR---------------------------SIAFRGDEFKEREEAFESQYVKQKERDDLKRMAEKKK 59
 S. cerevisiae   1 MLP--------RSALARSLQL-------QRGVAAR------FYSEGST-----------GTPRGSGSEDSFVKRERATEDFFVRQREKEQLRHLKEQLE 67
    H. sapiens   1 M-----------AVTALAARTWLGVW-GVRTMQARG-----FGSDQSENVDR-------GAGSIREAGGAFGKREQAEEERYFRAQSREQLAALKKHHE 75
      B. tarus   1 M-----------AATALAARTRQAVW-SVWAMQGRG-----FGSESGDNVRS-------SAGAVRDAGGAFGKREQAEEERYFRARAKEQLAALKKHHE 75
     S. scrofa   1 M-----------AATALAVRSRIGAW-SVWAMQSRG-----FSSDTPEGVRS-------GAGAVRDAGGAFGKKEQADEERYFRARAREQLAALKKHHE 75
    C. elegans   1 M-----------LSVSRAATR-------MTGMVAR------FSAGGHG---DGAGRGGGSGGSIRDAGGAFGKMEAAREDEYFYKKQKAQLQELREHIQ 72
     T. gondii   1 MSS-PCC-----VAIRRVARTTLES--GRRQVDSKSTDVSPFFTGTQQ--MSLPSAGMVTKIRNFSSVKFMDQKRSGEETVYFKKEDEALLRNLLANHP 89
     T. brucei   1 MLP---------LRVLPFIAA------GGYASQLR------FYSEGKP---TEGHRKINLDDDERWIEAEFDEKLRSPEERYALERQRETMKGLIKKIR 75
    E. gracilis  1 MAA-ACA--VRGFTTARPMLT-----PNKVKVPGR------------------------KPQDEEDLTWAEADRKLTPEERYARDKQMALLDKMTSQVE 67
    A. thaliana  1 M------------ATRNALRI---------------------------------------VSRRFSSGKVLSEEERAAENVFIKKMEQEKLQKLARQGP 48

 A. castellanii 63 IQENQP------IPETVVSQVSS--SSSSLNPFAQRAQSQQPSSSSHNDEPLTR--------GEFLEFRREIVQRLRNLEDD--IMDLQTR------RK 137
 D. discoideum  60 QEETKKAGSQP-TPNASSSANNS--KAAKIKEIEDK-----------------------------ISKLQKELADLKK--------------------- 105
 S. cerevisiae  68 KQ------------------------RKKIDSLENK-----------------------------IDSMTK---------------------------- 85
    H. sapiens  76 EEIVHH--------------------KKEIERLQKE-----------------------------IERHKQKIKMLKHDD------------------- 106
      B. tarus  76 NEISHH--------------------AKEIERLQKE-----------------------------IERHKQSIKKLKQSEDDD---------------- 109
     S. scrofa  76 NEISHH--------------------VKEIERLQKE-----------------------------IERHKQSIKKLKNDDDD----------------- 108
    C. elegans  73 EEVKHH--------------------EGQLENHKKV-----------------------------LERHQQRISEIEAQERALG-------------KE 109
     T. gondii  90 EYDPKYSVDHM---------------NAEVGSIARD---------------IT------------LACQKHGMKDPSAAFMKDLISIFGAHGYAKN-SK 145
     T. brucei  76 NEHKQH----------MHESVRE--RDAQIDNLKEQ-----------------------------IQTMEKKLQQLTNEKQ------------------ 115
    E. gracilis 68 ELEKSH----------TEQKKSNKGVKAQIEAISRQ-----------------------------LEALKAQLKE------------------------ 103
    A. thaliana 49 GEQAAGSASEAKVAGATASASAE--SGPKVSEDKNR-----------NYAVVAGVVAIVGSIGWYLKAGGKKQPEVQE--------------------- 113
```

Figure S7. Multiple sequence alignment of IF1 from various eukaryotes, related to Figure 2.

An alignment of IF1 from *Acanthamoeba castellanii* (ACANB08262), *Dictyostelium discoideum* (UniProt ID: Q9GSE7), *Saccharomyces cerevisiae* (UniProt ID: P01097), *Homo sapiens* (UniProt ID: Q9UII2), *Bos taurus* (UniProt ID: P01096), *Sus scrofa* (UniProt ID: Q29307), *Caenorhabditis elegans* (UniProt ID: O44441), *Toxoplasma gondii* (UniProt ID: A0A125YJP3), *Trypanosoma brucei* (UniProt ID: D0A1Z3), *Euglena gracilis* (UniProt ID: UPI0012B67DB9), and *Arabidopsis thaliana* (UniProt ID: Q9ZUX4).

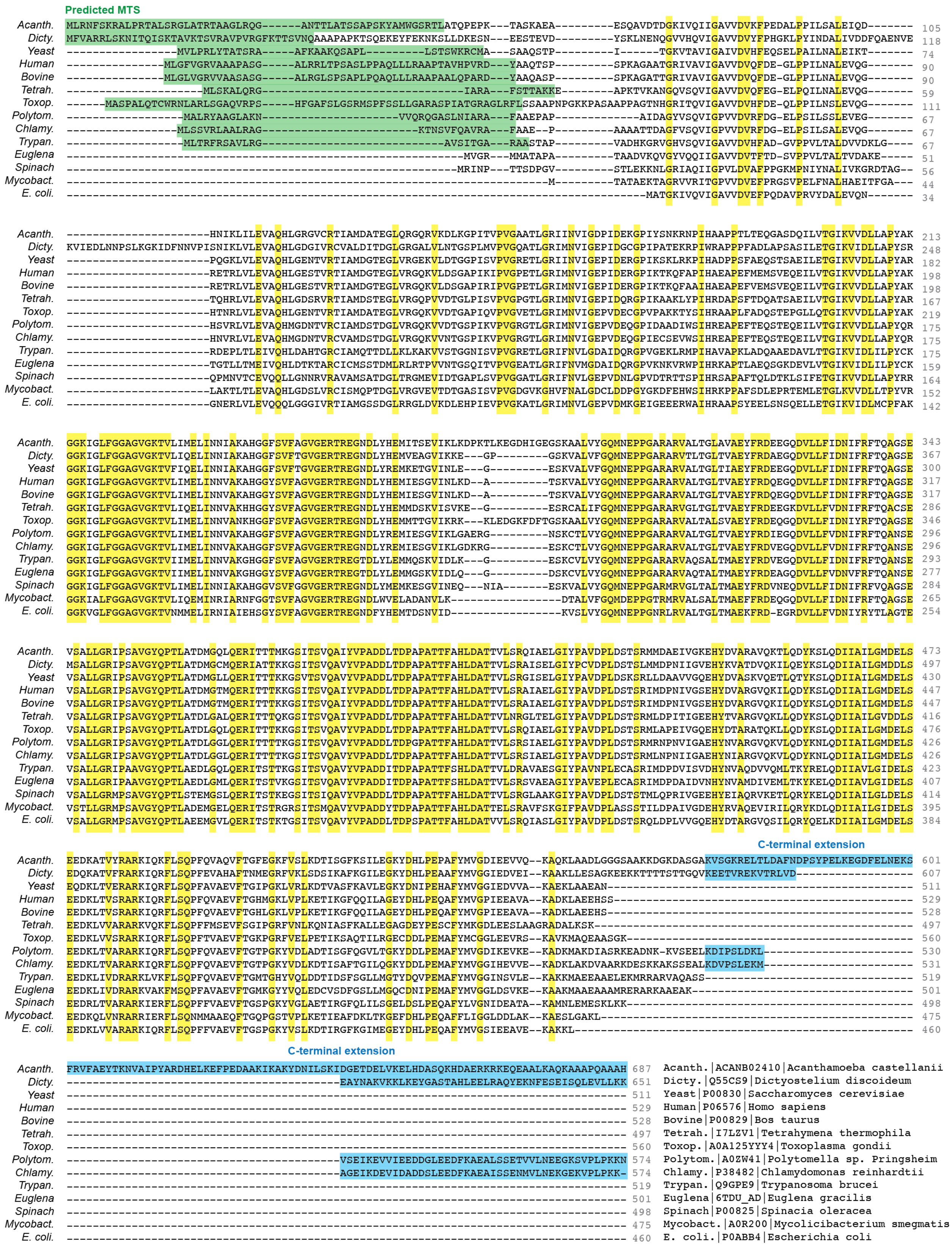

Figure S8. Multiple sequence alignment of the β subunit from various eukaryotes, related to Figure 3.

An alignment of β subunits from *A. castellanii*, *D. discoideum*, *S. cerevisiae*, *H. sapiens*, *B. taurus*, *T. thermophila*, *T.*

gondii, *Polytomella*, *C. reinhardtii*, *T. brucei*, *E. gracilis*, *S. oleracea*, *M. smegmatis*, and *E. coli*. Predicted mitochondria-targeting sequences (MTSs) (*green*) and C-terminal extensions (*blue*) are annotated. Conserved residues are highlighted in *yellow*.

A

```
Acanth.      --------------------------------------------------------------------------------------------
Dicty.     1 -------------------------------------------------------------------------MKN----------IVK---   6
Yeast        --------------------------------------------------------------------------------------------
Human      1 ----------------------------------MQTAGALFISPALIRCCTRGLIRPVSASFLNS--PVNSSKQPSYSNFPLQVARRE---  53
Bovine     1 ----------------------------------MQTTGALLISPALIRSCTRGLIRPVSASFLSR--PEIQSVQPSYSSGPLQVARRE---  53
Tetrah.      --------------------------------------------------------------------------------------------
Toxop.     1 MFFSRLSLSALKAAPAR---EALPGL---LSRQSFSSAGFSQFSSQKFF--FSPSRNFSQSPLFQKHTPVHCNQRIASALVPTQQPAMTRQN  84
Polytom.   1 -------------------------------------MSVQRLSLGAARCLSAGVARVQASQALVAQKAV-AVAPTRA-----QAAPAE---  46
Trypan.    1 -------------------------MMRRLAIQ-------------------SSVRRT-----TVAITPIAVPMKVAS-----PMCSAA---  35
Euglena    1 ------------------------------------------------------MQRGSSITKVVRRAALA---------------RST---  20
Spinach      --------------------------------------------------------------------------------------------
Mycobact.    --------------------------------------------------------------------------------------------
E. coli.     --------------------------------------------------------------------------------------------

Acanth.    1 ---MKNL-EIILQSSKMIGSGLAT-SGLIGAGAGVGIVFGCLILAFSRNPNLQKELFSYALIGFALTEAIGLLALVMAFLILFI-----  79
Dicty.     7 IEQLELA-SAVVELGKKVGAGLAA-IGLTGAGAGVGIVFAAFILAVGMNPNLRGELFKLAMLGFALSEAVGLLALMMSFLILYS-----  88
Yeast      1 --------MQLVLAAKYIGAGIST-IGLLGAGIGIAIVFAALINGVSRNPSIKDTVFPMAILGFALSEATGLFCLMVSFLLLFGV----  76
Human     54 FQTSVVS-RDIDTAAKFIGAGAAT-VGVAGSGAGIGTVFGSLIIGYARNPSLKQQLFSYAILGFALSEAMGLFCLMVAFLILFAM---- 136
Bovine    54 FQTSVVS-RDIDTAAKFIGAGAAT-VGVAGSGAGIGTVFGSLIIGYARNPSLKQQLFSYAILGFALSEAMGLFCLMVAFLILFAM---- 136
Tetrah.    1 --------MLLVKAVKVLVMGGCM-LPIAFGALGTGVLFAGFNVALSRNPEETESLFNNTLMGFALIETFIFMSIGLGFFVLFAA----  76
Toxop.    85 PYAMQVG-ARYDAGVASLSAAIAL-MSVGGVAQGIGSLFAALVSGTARNPSIKEDLFTYTLIGMGFLEFLGIICVLMSAVLLYS----- 166
Polytom.  47 VAQ-VRS-MSVLAASKMVGAGCAT-IALAGVGAGLGVMFGSLINGAARNPNIAKQLVGYALLGFALTESIALFSLLVVFLILFA----- 127
Trypan.   36 TRQASTV-AISVQGLHYVGTGLAA-IALAGVGLGIGTIFGNLLVACARQPNLTKMLFNYAILGFALTEAIGLFALMLAFLMLFS----- 117
Euglena   21 RNAAIAY-EVTVNGANLIGAGMAA-SGVGVPAIGVAMCFSSYMLAAARQPNMSAKLLPYCILGFALSEALALFTLLIALLELFVFS--- 104
Spinach    1 -------MNPLIAAASVIAAGLAVGLASIGPGVGQGTAAGQAVEGIARQPEAEGKIRGTLLLSLAFMEALTIYGLVVALALLFANPFV-  81
Mycobact.  1 ---MDLDPNAIITAGALIGGGLIMGGGAIGAGIGDGIAGNALISGIARQPEAQGRLFTPFFITVGLVEAAYFINLAFMALFVFATPGLQ  86
E. coli.   1 -------MENLNMDLLYMAAAVMMGLAAIGAAIGIGILGGKFLEGAARQPDLIPLLRTQFFIVMGLVDAIPMIAVGLGLYVMFAVA---  79
```

B

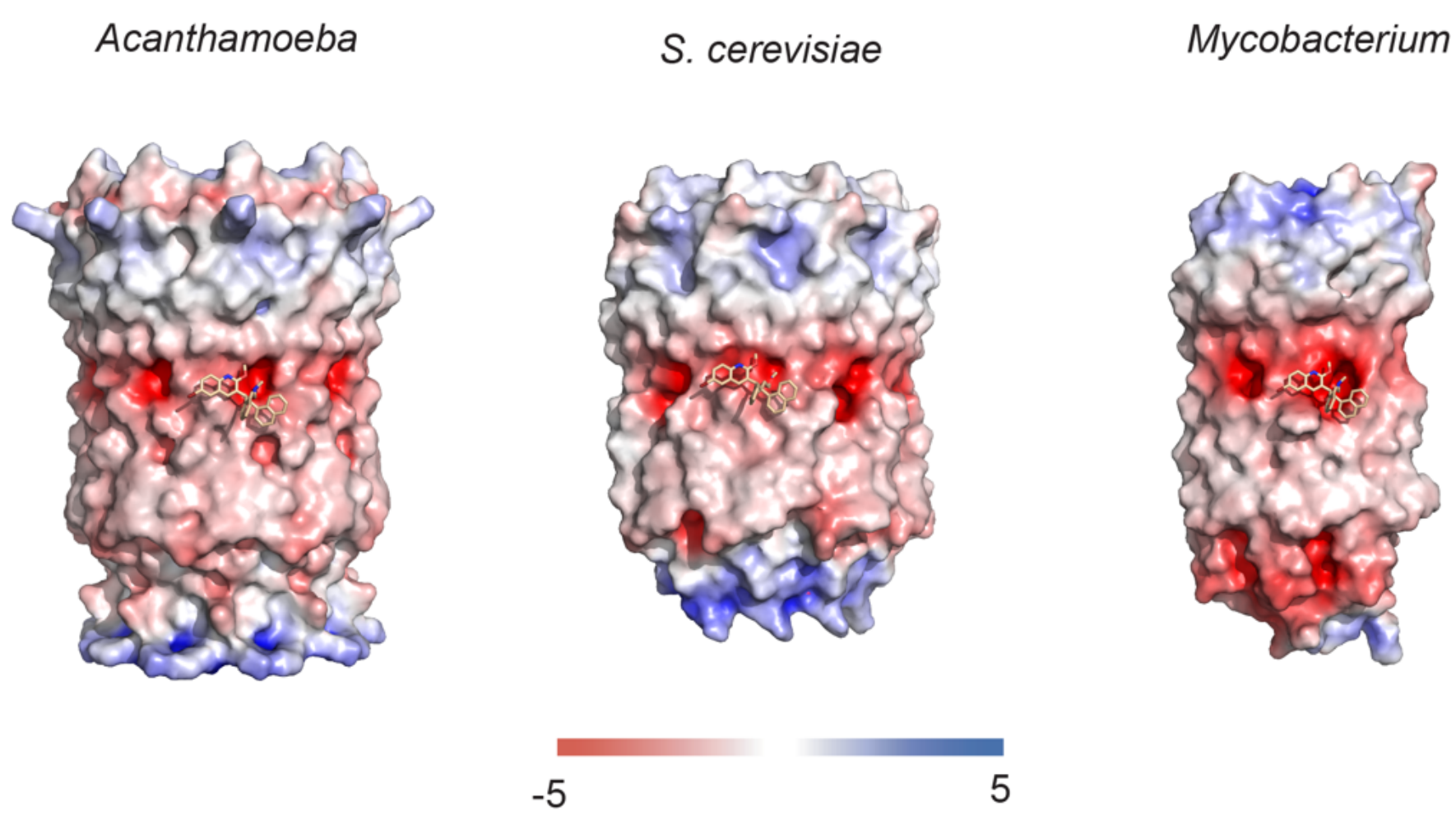

Figure S9. The c subunit in various eukaryotes, related to Figure 1.

(A) An alignment of subunit c from *A. castellanii*, *D. discoideum* (UniProt ID: Q37315), *S. cerevisiae* (UniProt ID: P61829), *H. sapiens* (UniProt ID: P05496), *B. taurus* (UniProt ID: P32876), *T. thermophila* (UniProt ID: Q951A5), *T. gondii* (UniProt ID: A0A125YJV2), *Polytomella* (UniProt ID: D7P7X5), *T. brucei* (UniProt ID: Q38C84), *E. gracilis* (PDB ID: 6TDU_AO), *S. oleracea* (chloroplast ATP synthase, UniProt ID: P69447), *M. smegmatis* (UniProt ID: Q5TIX5), and *E. coli* (UniProt ID: P68699). Conserved glutamate that binds to a proton is highlighted in *purple*. Conserved residues are highlighted in *yellow*.

(B) Surface representation of the c-subunit from *Acanthamoeba*, *S. cerevisiae* (PDB: 5BQ6), and *Mycobacterium* (PDB: 4V1F) colored by electrostatic charge (*red* (negative) to *blue* (positive)). A BDQ molecule is superimposed on the *Acanthamoeba* and yeast structure based on the binding site from the *Mycobacterium* structure.

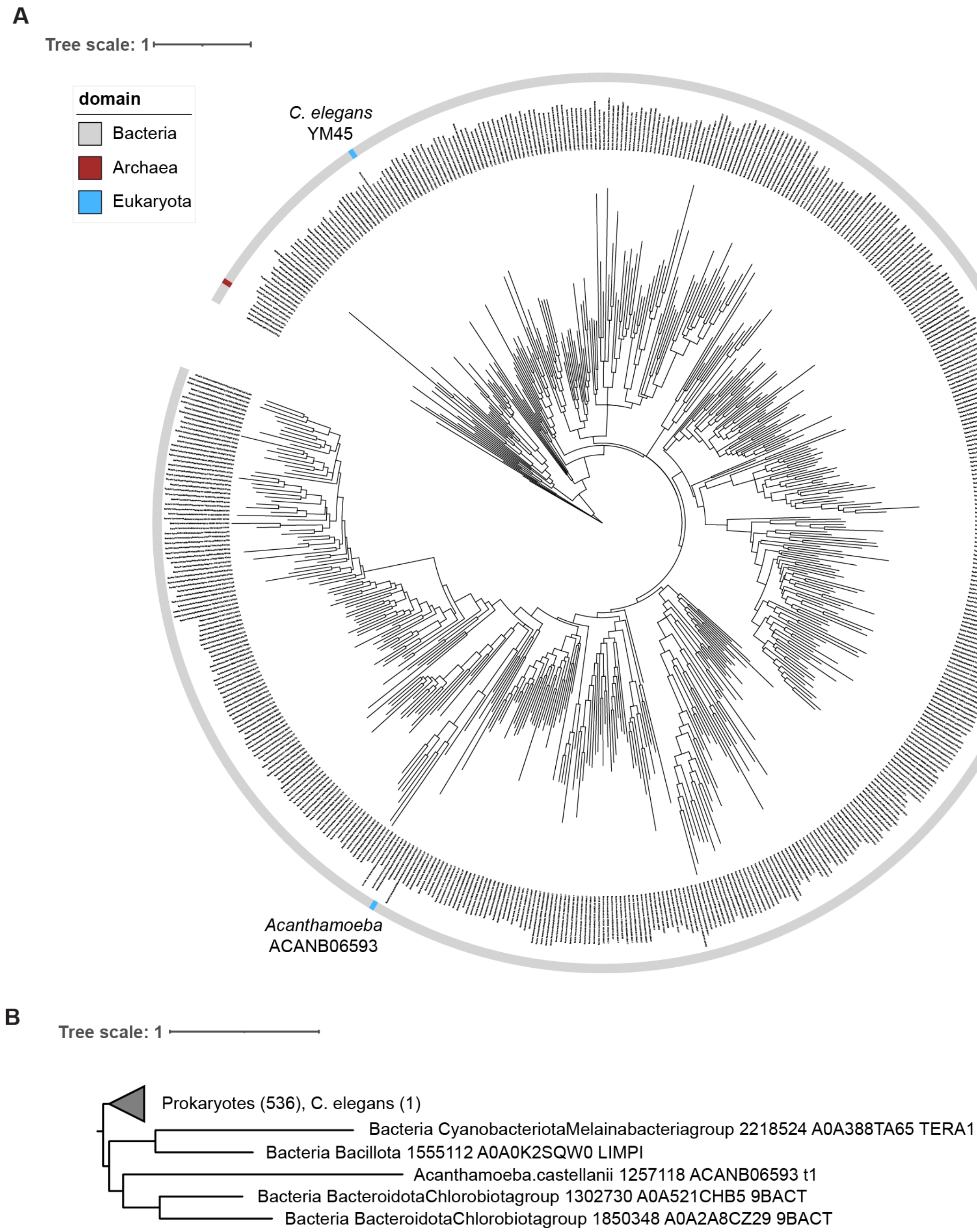

Figure S10. Subunit e in *Acanthamoeba* is the product of recent horizontal gene transfer from prokaryotes, related to Figure 4.

(A) Protein phylogeny of subunit e orthogroup members reveals two independent horizontal gene transfer (HGT) events from prokaryotes to eukaryotes, once to *Acanthamoeba* (ACANB06593) and once to *Caenorhabditis elegans* (*C. elegans* YM45). Colored ring indicates the domain membership of each leaf in the phylogeny: gray = bacteria, red = archaea, blue = eukaryote.

(B) A collapsed view of protein phylogeny of subunit e from (A) focusing on *Acanthamoeba* subunit e and its close prokaryotic neighbors.

```
Acanth.     1 -------------------MYASRSSGMACRSRLASRVAPRMGNAARLVSARG-FSQTLVTQQKKGEATTAAPVFKIDEEFDD----YKDVTASNEQVEKNYREPPVQVTSGKH  90
Dicty.      1 MLARFSRTAIKSGRYFSTSAKNTAPVSEAEE------IE-----ILKAVEAPQ-FKDAVKK---------------TRQQYQDMISKLTNDESGKSITQSTGLRPTIN-ISTPS  86
Yeast       1 ----------------------------------------------------M-FNRVFTR------------------SFAS-----SLRAA----ASKAAAPPPVR-LFGVE  33
Human       1 ---------------------------------------------MAAPAVSG-LSR-QVR------------------CFST-----SVV-R----PFAKLVRPPVQ-VYGIE  38
Bovine      1 --------------------------------------------------------------------------------------------------FAKLVRPPVQ-IYGIE  15
Tetrah.     1 -----------------------------------------MQVIRKHLAQKGLFNPFLFN------------------RFST----QQDI------TSVPGDKPPAI-EDSIH  44
Toxop.      1 ----MALPLLASRRLFSSFVFRGQPSTLSSNLSL-VRIRGLHG-GSLSPPSAT-LPR-AVQ------------------LFSS-----RIA-F----STA-AAEDSGA-SQTLE  76
Polytom.    1 --------------------------------ML-ARVASV---ALRRAEGKI-MPQMV-R------------------AL-------SVSAASAAQAELKLPTAPLQ-LSGTS  50
Trypan.     1 -------------------MFRRLSS--SAR--------AVVA-ARFYTPPEG-----L------------------KKLYAS--------------DF-ENSKYPLNIVPSDS  46
Euglena     1 -----------------MHMRRAVSVFGRCR--------SLNGLRNYAVPSPK-----Y------------------IEIYQS--------------DF-SRNAYPLELLGGSH  51

Acanth.    91 GEFASTLYATASV--LNAVEDVERDLKWLVDASQTIPMFQQFLSSGLPDKKVMRKLLQGVLK-GADFHRLTQKFLVTELLEAGELKLVPDIAAVYAQIMKAHRKEVPVVFTFAS 201
Dicty.     87 GKIAHALFDAASS--MRSVGIVAKELTQVKTIIDKTPGLKTALEGD-IDNSEKTVML-ELLQGSVTLSPVSGFF-LYYMTYENNFKLINPALTDFKRLVGSLDTEMSIKLTVAH 195
Yeast      34 GTYATALYQAAAK--NSSIDAAFQSLQKVESTVKKNPKLGHLLLNPALSLKDRNSVIDAIVETHKNLDGYVVN-LLKVLSENNRLGCFEKIASDFGVLNDAHNGLLKGTVTSAE 144
Human      39 GRYATALYSAASK--QNKLEQVEKELLRVAQILKEPKVA-ASVLNPYVKRSIKVKSLNDITA-KERFSPLTTN-LINLLAENGRLSNTQGVVSAFSTMMSVHRGEVPCTVTSAS 147
Bovine     16 GRYATALYSAASK--QNKLEQVEKELLRVGQILKEPKMA-ASLLNPYVKRSVKVKSLSDMTA-KEKFSPLTSN-LINLLAENGRLTNTPAVISAFSTMMSVHRGEVPCTVTTAS 124
Tetrah.    45 GKYAGVLFSSASS--NKSLNKVAEDMKYFNQLYKESEVFKSFLNNVSLKRNQQRDIISALGK--TNFNPATNN-LLETLIENKRLDSLPKIAEKYMDYYRILNKQESITIISAQ 153
Toxop.     77 GRYASALFRVAKK--KNQLEKVYGDLESVRNALKDSSEFRLFVDSPAVSVQQKLDVLRQLVN-RYKFDPLTGN-LLTTLVENKRLPMLARVADAFDAMYRKEKGEVKCLVTSAK 186
Polytom.   54 GAIATLAWQVAAK--ENVLAKVQDELYQLVEVFKSHPEIRRLATDPFLPDAFRRKVVRDMFA-TKDVTEVTKR-LVEALAEENSLSAIVQVTLAYEELMLAHKKEVHCTVVTAQ 163
Trypan.    47 VLFAKFLYKAAEE--KGNFDNILSDFQKIAAAASKLPIFW-----------ERTAVVEKIPE-FKQLSEPTF-FTLVWMQNNGMLELIQEVAEVYETFVNAKQKKAVAKIFVAP 145
Euglena    52 VDFAKLLYSFADQVENKKFEVYVEDFKKLDSIIAEKGPFW-----------AEEKIF-QSPT-FQGLSEGFK-FILGWIQSEGAIDRLENVRLAYKELVNEARKETTATVIVAK 151

Acanth.   202 LPDKDFLTRQIERVK----KYRLEADATPMWEFKINPALIGGFTVEVGSNYKGNFALSSQFDLMRQQLK---------------QAEDKWERVRPKE--LHINSFDTSSLFAPL 294
Dicty.    196 QYSEAEKKDLETNVK-----SFFPPETQFKFSYNVDPEIQKGYIIESPFINHDAS-YSTAVKKVKAQEQSVLAEFLNDIKNGIKNQTAVWETKEFRDKYLTLDESKN------- 296
Yeast     145 PLDPKSFKRIEKALSA---SKLVGQGKSLKLENVVKPEIKGGLIVELGDKTVDLS-ISTKIQKLNKVLE---------------DSI--------------------------- 212
Human     148 PLEEATLSELKTVLK-----SFLSQGQVLKLEAKTDPSILGGMIVRIGEKYVDMS-VKTKIQKLGRAMR---------------EIV--------------------------- 213
Bovine    125 ALDEATLTELKTVLK-----SFLSKGQVLKLEVKIDPSIMGGMIVRIGEKYVDMS-AKTKIQKLSRAMR---------------EIL--------------------------- 190
Tetrah.   154 ELTAAEKQKVEQGLK------KGNANVQFTVVYQVDPAILGGLQMYSGNNFLDCS-LLSRVNKLKTEIA---------------KISF-------------------------- 219
Toxop.    187 PLSAQQQKEIVAALQ-----NRAGTQARLIIDYAVSPQIMGGLVVRLGEQVLDFS-VATRLDRLQSQLL---------------APL--------------------------- 252
Polytom.  161 PLDKLERVELTKKAE-----KFVDAGFKLVMQEKIDKKLLGGFVIEFSDRRVDMS-TAKKVEEFNNFVN---------------KLVLSI------------------------ 229
Trypan.   146 GG-EKNVEEARRVAEELHKGLKELADYTLVLKTVVDRTIVKGFAVELAGQYVNKAEGQQ------KQAG--------------RADEVDYTNL-----------------PAPK 221
Euglena   152 EPSGNDLAEIRKQVEELHKE-SPLKDYKLVLETKVDPSIGGGYILEVCNQVVNRSAAAAAAETAALAKA--------------SAAQVDWTSL-----------------PAAP 233

Acanth.   295 SGVKITAADLDKTLADVLGSKKEAFQAADLDRTLKQALEDAQRDAGFSGDYEKNVLEQTRNLDFEKLKTVKQLDLAQHEKHLMQKHNIADNDAEGKRKVKAKIEELKRFKELDT 408
Dicty.        ------------------------------------------------------------------------------------------------------------------
Yeast         ------------------------------------------------------------------------------------------------------------------
Human         ------------------------------------------------------------------------------------------------------------------
Bovine        ------------------------------------------------------------------------------------------------------------------
Tetrah.       ------------------------------------------------------------------------------------------------------------------
Toxop.        ------------------------------------------------------------------------------------------------------------------
Polytom.      ------------------------------------------------------------------------------------------------------------------
Trypan.   222 PQKTVW---DDNIETEVLRKYLDGLSQYDMEEAKYGV----------------------------------------------------------------------------- 255
Euglena   234 PRPSPS---APDTLIRLLGSVVDDLADADKVEQKYGA----------------------------------------------------------------------------- 267

Acanth.   409 SFIGEVLKKHQPETYKKYQSVFQ  431
Dicty.        -----------------------
Yeast         -----------------------
Human         -----------------------
Bovine        -----------------------
Tetrah.       -----------------------
Toxop.        -----------------------
Polytom.      -----------------------
Trypan.       -----------------------
Euglena       -----------------------
```

Figure S11. Multiple sequence alignment of OSCP from various eukaryotes, related to Figure 1.
An alignment of OSCP from *A. castellanii*, *D. discoideum*, *S. cerevisiae*, *H. sapiens* (UniProt ID: P18859), *B. taurus* (UniProt ID: P02721), *T. thermophila* (UniProt ID: I7MMI7), *T. gondii* (UniProt ID: A0A125YKF8), *Polytomella* (UniProt ID: D8V7I1), *T. brucei* (UniProt ID: Q38AG1), and *E. gracilis* (PDB ID: 6TDU_WA). Conserved residues are highlighted in *yellow*.

```
                                 Predicted MTS                         N-terminal extension
Acanth. MDH (mito) ACANB11954    MRRGLSTVSRSATVFGKQACLASSTLRVDRRAYATSPEDAALEAQIEKVLDLVKKQGNLGSEKLTVRKLTELRQTVPEIDRALSSVGDSAVKYMAELEKPGAGPLSAVPP   110
Dicty. MDHB (mito) Q54D04        --------------------------------------------------------------------------------------MNKILTRSFSTGANFFASGKGKAP   24
Human MDH2 (mito) P40926         --------------------------------------------------------------------------------------MLSALARPASAALRRSFSTSAQNN   24
Yeast MDH (mito) P17505          ---------------------------------------------------------------------------------------------MLSRVAKRAFSSTVANP   17
Acanth. (non-mito) ACANB03251    ---------------------------------------------------------------MSADSNATTRRAVSRMSRIAAHMGVGSQTGFEAAATSASRVVSQQPP   47
Acanth. (non-mito) ACANB12424   ----------------------------------------------------------------------------------------------------------MAEP   4
Human MDH1 (Cyto) P40925         ----------------------------------------------------------------------------------------------------------MSEP   4
Yeast MDH (Cyto) P22133          -------------------------------------------------------------------------------------------------MPHSVTPSIEQDS   13
Yeast MDH (Peroxisome) P32419    -------------------------------------------------------------------------------------------------------------M   1
Dicty. (non-mito) MDHA Q54GE6    -------------------------------------------------MEQAILRINNISSQLTGKCPFKAHGGGAGKCPYKLGGGQILDQSKAWSLVNVTANVDQVTP   61
Dicty. (non-mito) MDHC Q54VM2    ---------------------------------------------------------------------------------------------------------MSNEV   5

Acanth. MDH (mito) ACANB11954    VRVTVTGAAGAIGYAMLFRIASGEMLGKHQP------VILQLLELEPAMKALEGVIMELKDCAFP-LLHGVTA--SSDVNKAFEGADFAMLVGAKPRTKGMERGDLLKEN   211
Dicty. MDHB (mito) Q54D04        VRVAITGASGQIGYQLLFRIASGDMLGKDQP------IILQCLELPGAMNSLKGVSMELDDCAFP-LLKGIVQ--SDKPEEAFAGADYALLVGARPRSKGMERGDLLKAN   125
Human MDH2 (mito) P40926         AKVAVLGASGGIGQPLSLLLKNSPLV-----------SRLTLYDIAHTP----GVAADLSHIETKAAVKGYLG--PEQLPDCLKGCDVVVIPAGVPRKPGMTRDDLFNTN   117
Yeast MDH (mito) P17505          YKVTVLGAGGGIGQPLSLLLKLNHKV-----------TDLRLYDLKGAK----GVATDLSHIPTNSVVKGFTPEEPDGLNNALKDTDMVLIPAGVPRKPGMTRDDLFAIN   112
Acanth. (non-mito) ACANB03251    VTVLVTGAAGQIAYSLVFLVAQGRLLGEETP------IKLHLLDLPQFVGALQGLAMELEDCAFP-LLRGVVC--TGDLKEAFKGVDVALLVGSFPRQAGMERNDLLAKN   148
Acanth. (non-mito) ACANB12424   VRVLVTGAAGQIAYSLIFAIARGDMFGKDQP------VILHLLDIAPMIESLKGVVMEIEDTASP-LVHGVVA--TTDLQEAFTNINYAILVGAMPRKEGMERKDLLKAN   105
Human MDH1 (Cyto) P40925         IRVLVTGAAGQIAYSLLYSIGNGSVFGKDQP------IILVLLDITPMMGVLDGVLMELQDCALP-LLKDVIA--TDKEDVAFKDLDVAILVGSMPRREGMERKDLLKAN   105
Yeast MDH (Cyto) P22133          LKIAILGAAGGIGQSLSLLLKAQLQYQLKESNRSVTHIHLALYDVNQ--EAINGVTADLSHIDTPISVSSHSP--AGGIENCLHNASIVVIPAGVPRKPGMTRDDLFNVN   119
Yeast MDH (Peroxisome) P32419    VKVAILGASGGVGQPLSLLLKLSPYV-----------SELALYDIRAAE----GIGKDLSHINTNSSCVGYD---KDSIENTLSNAQVVLIPAGVPRKPGLTRDDLFKMN   93
Dicty. (non-mito) MDHA Q54GE6    IKVLVTGAAGQIAYSLMFMIASGQMFGPHQP------VILHLLDIPKMADALKGVVMELQDCSYP-LLQSVVA--TTDIQTAFLHINVAILVGAFPRGPGMQRKDLLKVN   162
Dicty. (non-mito) MDHC Q54VM2    INVLITGAAGQIAYSLIFNVCKGDMFGLDQR------IKLHLLDIPQMVDSLKGIVMEIQDGAYP-LIADTVI--TADVKEAFTGVHYAILVGAMPRREGMERADLLKAN   106

Acanth. MDH (mito) ACANB11954    ANIFSVQGKALNKVANRDTLRVCVVGNPANTNALI----ASANAPNIH----PRRFTAMTRLDHNRGIAQLAD---KLKCKVTDI-----------ERFAIWGNHSATQY   299
Dicty. MDHB (mito) Q54D04        AEIFSVQGKALDKSANRDTLRVLVVGNPANTNALI----AARNAPNID----PKRFSAMTRLDHNRGLAQLAD---KTGSAVTDI-----------EKFCIWGNHSATQY   213
Human MDH2 (mito) P40926         ATIVATLTAACAQHC--PEAMICVIANPVNSTIPI-TAEVFKKHGVYN----PNKIFGVTTLDIVRANTFVAE---LKGLDPARV-----------NVPVIGGHAGKTII   206
Yeast MDH (mito) P17505          ASIVRDLAAATAESA--PNAAILVISNPVNSTVPI-VAQVLKNKGVYN----PKKLFGVTTLDSIRAARFISE---VENTDPTQE-----------RVNVIGGHSGITII   201
Acanth. (non-mito) ACANB03251    AAIFKEQGRALDQYAS-KNVKVCVVGNPANTNALI----AQACAPSIP----KENFTALTRLDQNRAKGFLAK---KLHVSPGQI-----------HNAIIWGNHSSTQF   235
Acanth. (non-mito) ACANB12424   CGIFKVQGKALNDYAN-KDVKVLVVGNPANTNALI----AALSAPGLP----KENFSCLTRLDHNRAKSQISK---RLNINVNQV-----------HNVIIWGNHSKTQY   192
Human MDH1 (Cyto) P40925         VKIFKSQGAALDKYAK-KSVKVIVVGNPANTNCLT----ASKSAPSIP----KENFSCLTRLDHNRAKAQIAL---KLGVTANDV-----------KNVIIWGNHSSTQY   192
Yeast MDH (Cyto) P22133          AGIISQLGDSIAECCDLSKVFVLVISNPVNSLVPVMVSNILKNHPQSRNSGIERRIMGVTKLDIVRASTFLREINIESGLTPRVNSMP--------DVPVIGGHSGETII   221
Yeast MDH (Peroxisome) P32419    AGIVKSLVTAVGKFA--PNARILVISNPVNSLVPI-AVETLKKMGKFK----PGNVMGVTNLDLVRAETFLVD---YLMLKNPKIGQEQDKTTMHRKVTVIGGHSGETII   193
Dicty. (non-mito) MDHA Q54GE6    VSIFKEQGEALNKYAS-RGVKVLVVGNPANTNALT----ALMKASDLP----TSNFSALTRLDQNRAQSMISE---KVGTNVDNV-----------HNVIIWGNHSQTQV   249
Dicty. (non-mito) MDHC Q54VM2    AAIFKVQGKALAEHAN-KNVKVLVVGNPANTNALI----AQVSANGIP----KENFTCLTRLDQNRAKSQIAL---KAGVNVKDV-----------HNVIIWGNHSSTQY   193

Acanth. MDH (mito) ACANB11954    PDISHTQINGKW--------AKDLVDEKWVKDTFIPDVQQRGAAIIAARG------SSSAASAANAAIEHMRDWVKGT-NGQWTSMGVWTGNGKVG-DYGTSPDI-----   388
Dicty. MDHB (mito) Q54D04        PDINFGTVKGKS-------LVDTINDQKWVKDNFIPTVQQRGAAIIAARG------LSSAASAASAAIDHMRDWTYGT-NGQWTSMAIYSE----G-EYGADKGL-----   299
Human MDH2 (mito) P40926         PLISQCTPKVDF------------PQDQL--TALTGRIQEAGTEVVKAKAGAGSATLSMAYAGARFVFSLVDAMNGK----EGVVECSFVK-------SQETECT-----   286
Yeast MDH (mito) P17505          PLISQTNHKLMS-------------DDKR--HELIHRIQFGGDEVVKAKNGAGSATLSMAHAGAKFANAVLSGFKGE----RDVIEPSFVD----SPLFKSEGIE-----   283
Acanth. (non-mito) ACANB03251    PDVSHGQVSIDG---RTVSLKEAVGGTEWVEKEFIPGVQQRGAAVIKARQ------KSSAASAANAVVGHVHDWLVGTPQGEWVSMGVYSD----G-SYGIPPGV-----   326
Acanth. (non-mito) ACANB12424   PDVNHAYISGSATGGEKSQVRGAVADDAWLQGEFITTVQNRGGAIIAARK------LSSAASAAQAIVDHMRDWVKGTPEGEIVSMGVWSD----G-SYGVAKDL-----   286
Human MDH1 (Cyto) P40925         PDVNHAKVKLQG---KEVGVYEALKDDSWLKGEFVTTVQQRGAAVIKARK------LSSAMSAAKAICDHVRDIWFGTPEGEFVSMGVISD----GNSYGVPDDL-----   284
Yeast MDH (Cyto) P22133          PLFSQSNFLSRL------------NEDQL--KYLIHRVQYGGDEVVKAKNGKGSATLSMAHAGYKCVVQFVSLLLGNIEQIHGTYYVPLKD----ANNFPIAPGADQLLP   313
Yeast MDH (Peroxisome) P32419    PIITDKSLVFQL-------------DKQY--EHFIHRVQFGGDEIVKAKQGAGSATLSMAFAGAKFAEEVLRSFHNEKPETESLSAFVYLP----GLKNGKKAQQ---LV   281
Dicty. (non-mito) MDHA Q54GE6    PDVNHGYILNYPNRGLIEPIPSSVNDDKWLNEQFIPLVQNRGATVIAARK------LSSAASAANAIVGHVRDWLLGTKDGEHVSMAVYSD----G-SYNVPKGL-----   343
Dicty. (non-mito) MDHC Q54VM2    PDYRCGYINLST---GKTPISTAIKDEKWLQGEFISTVQKRGAAVIAARK------LSSAASAAKAITDHMHDWVLGTAEGEYVSMGVYSD----G-SYGVPEGL-----   284

Acanth. MDH (mito) ACANB11954    ------YYSFPVVCAD-G----EYTIVQNVPVDKFS---AERMQKTNDELVSEKNGVGELAKRTTDPAALLKEQYKGGKQ   454
Dicty. MDHB (mito) Q54D04        ------YFSFPVIVDNKG----KYEIVKGLKLDQFS---QERFDATRKELLSEMDGVKELLP------------------   348
Human MDH2 (mito) P40926         ------YFSTPLLL---G----KKGIEKNLGIGKVSSFEEKMISDAIPELKASIKKGEDFVKTLK---------------   338
Yeast MDH (mito) P17505          ------FFASPVTL---G----PDGIEKIHPIGELSSEEEEMLQKCKETLKKNIEKGVNFVASK----------------   334
Acanth. (non-mito) ACANB03251    ------IYSFPLTCSR-G----NWAIVQGLPVDAAA---RKRMQATYDELHSEKETAFSFLGLDKKH-------------   379
Acanth. (non-mito) ACANB12424   ------IYSFPVTCQD-G----KYTIVQGLDVDAFS---REKLELTEKELQEEKEEASAFLH------------------   334
Human MDH1 (Cyto) P40925         ------LYSFPVVIKN-K----TWKFVEGLPINDFS---REKMDLTAKELTEEKESAFEFLSSA----------------   334
Yeast MDH (Cyto) P22133          LVDGADYFAIPLTITTKGVSYVDYDIVN--RMNDME--RNQMLPICVSQLKKNIDKGLEFVASRSASS------------   377
Yeast MDH (Peroxisome) P32419    GDNSIEYFSLPIVLRN-G----SVVSIDTSVLEKLSPREEQLVNTAVKELRKNIEKGKSFILDSSKL-------------   343
Dicty. (non-mito) MDHA Q54GE6    ------IFSFPVTCHN-G----QWTIVQGLKINSST---QQKIDATIKELQEEKETAMSFLN------------------   391
Dicty. (non-mito) MDHC Q54VM2    ------IFSFPVKCAN-G----KYTIVQGLQMDDLS---KNLINLTTEELVAEKTTALQFLSE-----------------   333
```

Figure S12. Multiple sequence alignment of MDHs from various cellular compartments and eukaryotes, related to Figure 5.

An alignment of MDHs from *A. castellanii* (mitochondrial), *D. discoideum* (mitochondrial), *S. cerevisiae* (mitochondrial), *H. sapiens* (mitochondrial), *A. castellanii* (not mitochondrial), *A. castellanii* (not mitochondrial), *H. sapiens* (cytosolic), *S. cerevisiae* (cytosolic), *S. cerevisiae* (peroxisomal), *D. discoideum* (not mitochondrial), and *D. discoideum* (not mitochondrial). Predicted mitochondria-targeting sequences (MTSs) (*blue*) and N-terminal extensions (*grey)* are annotated. Conserved residues are highlighted in *yellow*.

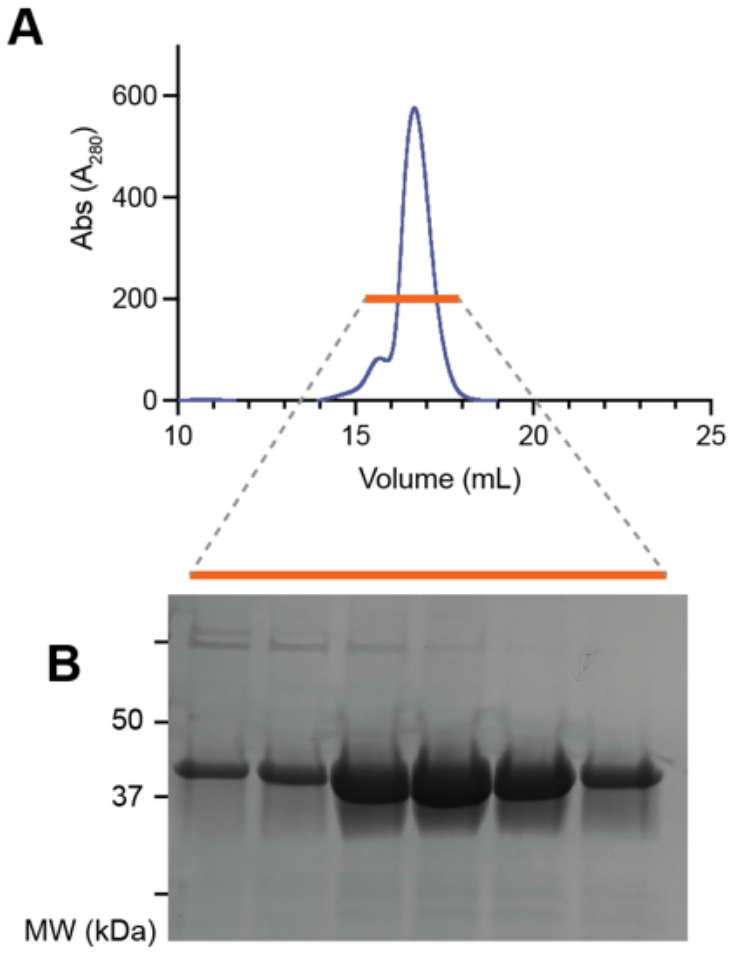

C

	apo AcMito-MDH
**Data collection**	
Space group	P1 2_1 1
Cell dimensions	
a, b, c (Å)	50.408, 136.498, 125.548
α, β, γ (Å)	90.0, 100.85, 90.0
Resolution (Å)	34.54 - 2.096 (1.12 - 2.1)
Wavelengngth (Å)	0.979110
R_{merge}	0.3569 (3.33)
R_{pim}	0.1499 (1.383)
I/σ	1.56 (2.10)
Completeness (%)	96.2 (94.11)
Redundancy (%)	6.5 (6.7)
**Refinement**	
Resolution (Å)	34.54 - 2.33
No. reflections	95755
R_{work}/R_{free} (%)	0.210/0.259
No. atoms	
Protein	10135
Solvent	300
B-factors	
Protein	27.9
Bond RMSD	
Lengths (Å)	0.009
Angles (°)	1.00
**Validation**	
MolProbity score	1.46
Clashscore	6
rotamer outliers (%)	0
Cβ outliers (%)	0
Ramachandran outliers (%)	0
Ramachandran favored (%)	98.04
Ramachandran allows (%)	1.96

*Values in parentheses are for the highest-resolution shell

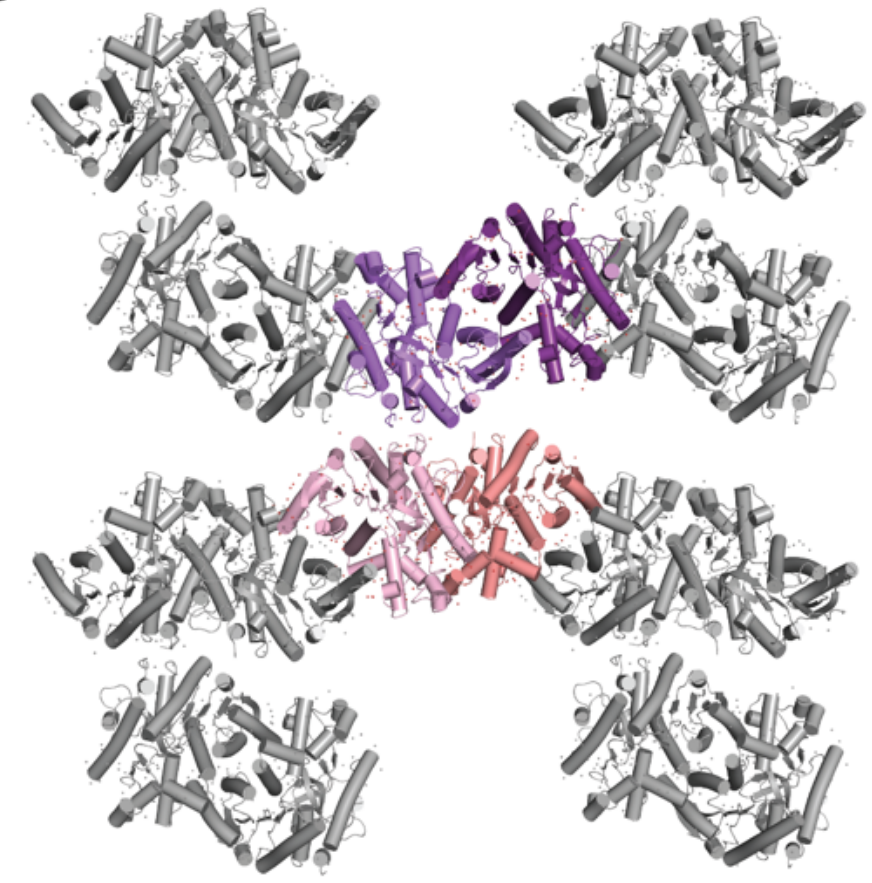

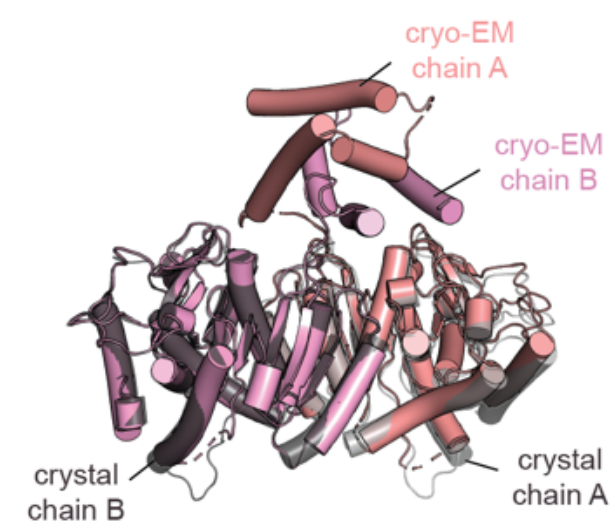

F

	recombinant MDH	AcATP synthase	AcATP synthase$_{MDH\Delta NTD}$	AcATP synthase$_{MDH\text{-}H294A}$	AcATP synthase + ATP	AcATP synthase + ADP
Best fit values						
V_{max} (μM/s)	0.4863	0.2452	0.4547	0.1296	0.2165	0.2812
K_M (μM)	58.56	19.73	53.81	n.d.	23.50	36.08
K_I (μM)	4232	8348	4595	n.d.	10931	8311
k_{cat} (apparent, s^{-1})	435.0	980.8			865.8	1125
95 % CI (profile likelihood)						
V_{max} (μM/s)	0.4489 - 0.5301	0.2264 - 0.2664	0.4087 - 0.5116	n.d.	0.2008 - 0.2339	0.2619 - 0.3028
K_M (μM)	46.06 - 74.26	13.03 - 28.51	38.34 - 74.94	n.d.	16.11 - 33.12	27.14 - 47.29
K_I (μM)	3204 - 5610	5765 - 12612	3114 - 6851	n.d.	7591 - 16655	6109 - 11627
k_{cat} (apparent, s^{-1})	394.4 - 483.9	905.6 - 1066			803.3 - 935.6	1048 - 1211

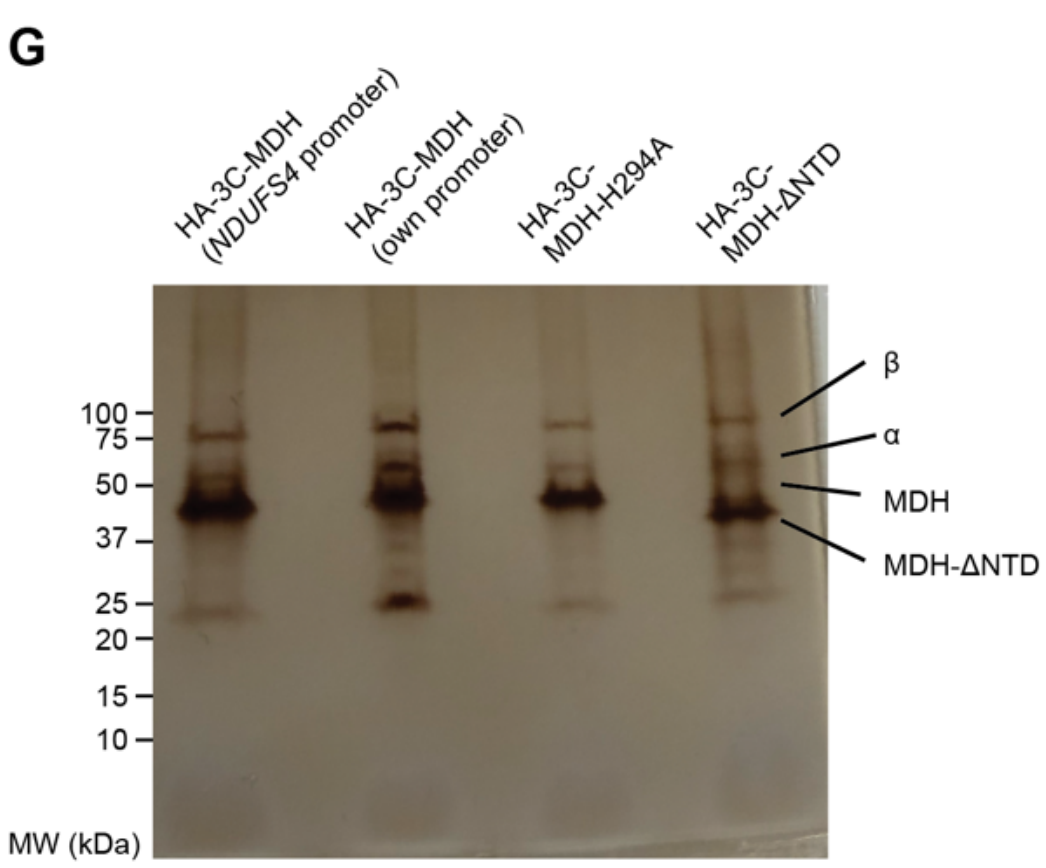

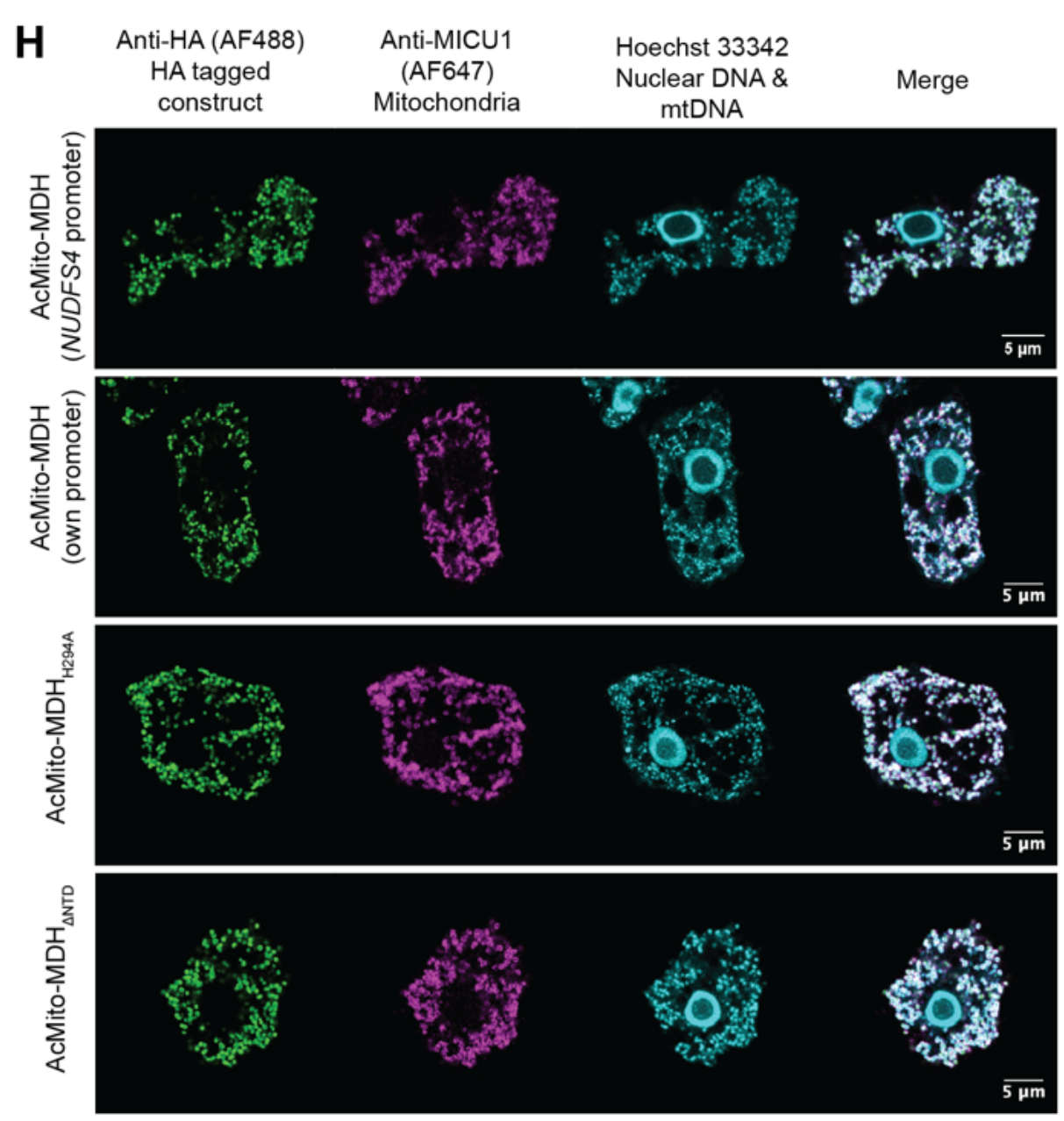

Figure S13. The crystal structure of Acmito-MDH, related to Figure 5.

(A) Size Exclusion Chromatogram trace of the absorbance at 280 nm of nickel eluate for AcMito-MDH.

(B) An SDS-PAGE gel of the peak highlighted by the orange line in (A).

(C) Data collection and refinement statistics for the AcMito-MDH crystal structure.

(D) The asymmetric unit of AcMito-MDH contains four distinct monomers. Two form a dimer with each other (*lilac* and *purple*) and two form a dimer with a symmetry mate (*salmon* and *pink*).

(E) An overlay of cartoons of the cryo-EM structure (*salmon* and *pink*) and the crystal structure (apo, *gray*).

(F) Kinetic constants of recombinantly expressed AcMito-MDH and endogenous AcATP synthase, AcATP synthase$_{MDH\Delta NTD}$, AcATP synthase$_{MDH\text{-}H294A}$ and AcATP synthase with ATP or ADP.

(G) A silver-stained SDS-PAGE gel of the purification of AcATP synthase with a HA-tagged MDH expressed under the *NDUFS4* promoter and its own promoter and a catalytically dead MDH in complex with AcATP synthase (AcATP synthase$_{MDH\text{-}H294A}$) and a ΔNTD-MDH in complex with ATP synthase (AcATP synthase$_{MDH\Delta NTD}$) both expressed under its own promoter.

(H) Confocal microscopy images validating localization to the mitochondria of the tagged constructs. Constructs were stained with an anti-HA antibody (*green*), mitochondria were stained by anti-MICU1 antibody (*magenta*), mitochondrial and nuclear DNA were stained by Hoescht 33342 (*cyan*).

REFERENCES

1. Punjani, A., Rubinstein, J.L., Fleet, D.J., and Brubaker, M.A. (2017). cryoSPARC: algorithms for rapid unsupervised cryo-EM structure determination. Nature Methods *14*, 290–296. https://doi.org/10.1038/nmeth.4169.
2. Liebschner, D., Afonine, P.V., Baker, M.L., Bunkóczi, G., Chen, V.B., Croll, T.I., Hintze, B., Hung, L.-W., Jain, S., McCoy, A.J., et al. (2019). Macromolecular structure determination using X-rays, neutrons and electrons: recent developments in ıt Phenix. Acta Crystallographica Section D *75*, 861–877. https://doi.org/10.1107/S2059798319011471.
3. Stefely, J.A., Deng, F.G., Chen, M.Z., Nguyen, K., Issa, T.M., Maliaris, K., Fry, M.Y., Luce, B.E., Rivera, K.D., Guo, X.A., et al. (co-submitted). Mitochondrial proteome and complexome of Acanthamoeba delineate aerobic and anaerobic pathways dynamically regulated by oxygen.
4. Jamali, K., Käll, L., Zhang, R., Brown, A., Kimanius, D., and Scheres, S.H.W. (2024). Automated model building and protein identification in cryo-EM maps. Nature *628*, 450–457. https://doi.org/10.1038/s41586-024-07215-4.
5. Abramson, J., Adler, J., Dunger, J., Evans, R., Green, T., Pritzel, A., Ronneberger, O., Willmore, L., Ballard, A.J., Bambrick, J., et al. (2024). Accurate structure prediction of biomolecular interactions with AlphaFold 3. Nature. https://doi.org/10.1038/s41586-024-07487-w.
